# Key Differences in the Gut Microbiota of Red-Claw Crayfish *Cherax quadricarinatus* with Different Sizes and Genders Under Consistent Farming Conditions

**DOI:** 10.3390/biology14091209

**Published:** 2025-09-07

**Authors:** Wen-Feng Li, An-Qi Zhao, Yan Chen, Zhao-Yang Yin, Yun-Xiang Mao, Zhe Qu, Shan Zhang, Hai Huang

**Affiliations:** 1 Yazhou Bay Innovation Institute, Hainan Tropical Ocean University, Sanya 572025, Chinayxmao@ouc.edu.cn (Y.-X.M.);; 2 Key Laboratory of Utilization and Conservation of Tropical Marine Bioresource, Ministry of Education, Hainan Tropical Ocean University, Sanya 572022, China; 3 Hainan Key Laboratory for Conservation and Utilization of Tropical Marine Fishery Resources, Hainan Tropical Ocean University, Sanya 572022, China; 4 College of Fisheries and Life Science, Hainan Tropical Ocean University, Sanya 572022, China; 5 Key Laboratory of Tropical Aquatic Germplasm of Hainan Province, Sanya Oceanographic Institution, Ocean University of China, Sanya 572025, China

**Keywords:** red-claw crayfish, *Cherax quadricarinatus*, intestinal microbiota, 16S rRNA sequencing, size, gender

## Abstract

The red-claw crayfish *Cherax quadricarinatus* has been widely introduced and cultured in China and has become a crucial economic freshwater species. However, individuals reared from the same batch of seedlings in uniform aquaculture systems exhibit significant size variation within and between genders, which notably impedes the optimization of both their quality and yield. Gut microbiota plays an important role in the metabolism, development, and immunity of aquatic animals. However, the knowledge on the intestinal microbiota of red-claw crayfish with various sizes and genders is poor. In this study, the intestinal microbiota of red-claw crayfish cultured in consistent farming conditions were separated to larger-sized female (GUBF), larger-sized male (GUBM), smaller-sized female (GUSF), and smaller-sized male (GUSM) groups based on their body size (weight) and gender, before being analyzed via high-throughput 16S rRNA gene sequencing. The intestinal microbiota results showed that alpha diversity tended to generally decrease in the order of GUBF, GUBM, GUSF, and GUSM, indicating that the richness and evenness of the gut flora were gradually improved with the increase in body weight or from male to female. Community richness and diversity were highest in the GUBF group, followed by the GUBM, GUSF, and GUSM groups, respectively. Beta diversity indicated significant differences in gut microbiota between the GUBF and GUSF, GUBM and GUSM, GUBF, and GUBM groups. Further analysis showed that the dominant phyla in the intestine of the red-claw crayfish were Firmicutes, Proteobacteria, Fusobacteriota, Bacteroidota, and Deinococcota, and the dominant genera were Vibrio, Tyzzerella, Candidatus Bacilloplasma, Citrobacter, and Candidatus Hepatoplasma. Moreover, nine phyla and 106 genera were identified to be significantly different in abundance among all four groups. Pairwise comparisons revealed that the phylum Dependentiae and Planctomycetota and genus Babeliaceae_unclassified were significantly abundant in the gut of female crayfishes, regardless of body size. On the other hand, irrespective of genders, the abundance of Novosphingobium, Piscinibacter, and Citrobacter was significantly increased or declined in the larger or smaller crayfishes, respectively. PICRUSt2 analysis based on the KEGG database suggested that the pathway bacterial secretion system, isoflavonoid biosynthesis, and pathway glycerolipid metabolism were significantly up- and down-regulated in female individuals, respectively, regardless of body sizes. Meanwhile, the adipocytokine signaling pathway, pyruvate metabolism, and pathway electron transfer carriers were significantly up- and down-regulated in larger individuals, respectively, regardless of gender. Gender differences may induce gut microbiota to exert a greater impact on hormonal regulation, whereas differences in individual size seem to lead gut microbiota to develop a preference for food intake and energy sources. In summary, this study revealed key differences in the intestinal microbiota of the crayfish with different sizes and genders, even in those which were cultured in the same environment and period, which potentially suggest that the intestinal microbiota may be influenced by some other factors in the culture system, such as hormone secretion, metabolism, and immunity. This study will contribute to improving growth performance and animal quality in the aquaculture of *C. quadricarinatus*.

## Simple Summary

As a high-commercial-value aquaculture species, the red-claw crayfish (*Cherax quadricarinatus*) has experienced continuous growth regarding aquaculture scale and production output. However, the significant size variation among individuals and genders from the same seedling batch, reared in consistent systems, hinders quality and yield. This variability is likely influenced by multiple factors, including genetics, sex, nutrition, and environmental conditions. The gut microbiota, crucial for nutrient acquisition and metabolism, may play a pivotal role. In this study, we analyzed the gut microbiota composition of crayfish with different body weights and sexes cultured in consistent aquaculture systems from the same batch of seedling. Our findings revealed significant variations in gut microbiota across groups differing in body weight and sex. These results are expected to provide valuable insights into the gut microflora associated with red-claw crayfish growth performance and provide a basis for future studies on the probiotic feed of *Cherax quadricarinatus*.

## 1. Introduction

As an important digestive and absorption organ, the gut typically harbors thousands of microbial species, and there are substantial variations in species’ relative abundances across individuals [1]. These gut microbial species mainly survive on the nutritional elements in the intestinal tract and are profoundly involved in a variety of physiological and biochemical functions of the host [2]. Generally, the relationships between intestinal microbial communities and their hosts usually provide important benefits to the hosts [3]: a rich, stable, and healthy microbial community in the intestines is essential for growth stimulation and reproduction in animals [4,5,6]. Furthermore, the structure and abundance of the microbial communities are closely linked to host metabolism, development, physiology, and health [7]. However, associations between the microbiota’s composition, function, and complex diseases have been observed in some cases, indicating that abnormal microbial communities may potentially trigger disease processes [1,8,9]. For example, the human gut microbiome has been linked to metabolic disease and obesity [9]. In the past few decades, the diverse and pivotal roles of intestinal microbiota across various organisms have garnered widespread recognition [2,10]. The continuously growing body of knowledge on the intestinal microbiome highlights the fact that the intestinal flora play key roles in facilitating digestion, nutrient absorption, innate immunity, and proliferation of epithelial cells and their structural and functional maturation, metabolism, and immunity [2,10,11].

As mentioned in previous reports, the intestinal microbiota is shaped by host-defined deterministic factors specified by the genotype. These deterministic factors include environmental factors such as diet and stochastic factors such as colonization order and history of antibiotic exposure [1]. Crustaceans, especially species that live in ocean water or freshwater for the majority of their lifespan, are exposed to higher microbial loads in the aquatic environment. The natural contact with the surrounding water may remarkably influence gut colonization, and water and feed become two main sources of microorganisms for the aquatic animals after they start feeding [11]. However, both the drivers of diversity and abundance in intestinal microbial community and the factors underlying the successful colonization and assembly of ingested microbes in the gut of crustaceans remain poorly understood. Furthermore, both the host genetics and gut microbiome could influence metabolic phenotypes, and the abundances of specific members of the gut microbiota are influenced in part by the genetic makeup of the host [12].

Plenty of bacteria reside in the intestinal tracts of aquatic animals, significantly influencing their phenotypes, including nutrient absorption, metabolism, energy consumption, development, immunity, and disease resistance [13]. On the other hand, host factors, such as dietary conditions, development level, and growth performance, also affect the intestinal bacteria composition [14]. The richness and evenness of intestinal microbiota usually increase with age in *Gymnocypris chilianensis*, *Pelteobagrus fulvidraco*, and *Ctenopharyngodon idellus* [15]. The intestinal flora of zebrafish (*Danio rerio*) and red swamp crayfish (*Procambarus clarkii*) varied in different developmental stages [16,17]. In the gut microbiota of grass carp (*Ctenopharyngodon idella*), the ratio of Bacteroidetes to Firmicutes and alpha diversity indices were related to the body mass of the hosts [18]. Lan et al. reported that the gut flora of giant freshwater prawn (*Macrobrachium rosenbergii*) differs among the high, medium, and low levels of growth performance groups [13]. In contrast, the structure of gut microbiota could affect the growth and development of the host organisms. Different gut microbiota affected the nutrient absorption and body weight of white-leg shrimp (*Litopenaeus vannamei*) [19], and the growth performance of sea cucumber (*Apostichopus japonicus*) was remarkably influenced by the microbial community of the intestine [20].

Furthermore, recent studies indicate that sexual dimorphism may be exerted or reinforced by host microbiota, with some sex-specific differences in gene expression and metabolism being driven by sex-specific differences in the microbiota [21]. In several species of fishes, such as *G. chilianensis* and *Coreius guichenoti*, the composition and structure of the intestinal microbiota changed between genders [15]. This also happens in zebrafish (*D. rerio*), yellow drum (*Nibea albiflora*), Siamese fighting fish (*Betta splendens Regan*), and cultured subadult pufferfish (*Takifugu obscurus*), where the composition and homeostasis of intestinal microbiota depends on gender [22]. Moreover, in the skin and mantle associated mucus communities of adult octopus males and females, a distinct microbial community composition in both were identified, which were hypothesized to be due to differences in hormone profile, as well as behavioral or ecological differences between sexes in the wild [21]. Moreover, gender bias is also evident in crustaceans. The core intestinal microbiota was significantly sex-biased in Chinese mitten crabs (*Eriocheir sinensis*) and mud crab (*Scylla paramamosain*) [22,23]. However, studies on the investigation of how host sex influences the microbiota of aquatic invertebrates are limited [24].

The red-claw crayfish *Cherax quadricarinatus* is a native species originally distributed across the northern tropics of Australia [25]. Due to its short breeding cycle, strong adaptability, and low susceptibility to diseases, it has become an economically important freshwater species farmed in many tropical and subtropical countries [25]. Though the red-claw crayfish was first introduced to China in 1992, a booming breeding has been set off due to a breakthrough in key seedling breeding technology in recent years [26]. It is now a promising aquaculture species cultured in many places [27], and the market demand for it has been continuously increasing due to its large body, high proportion of edible parts, high protein and low fat content, and rich amino acid content [25]. However, with the increasing demand for *C.quadricarinatus*, a supply bottleneck of genetically improved varieties has hindered the aquaculture industry. Consequently, breeding new strains with enhanced traits such as stress tolerance, high growth rates, and low feed conversion ratios is urgently needed to support this industry [27].

It is commonly observed that there are significant individual size differences among cultured red-claw crayfishes derived from the larvae of similar sizes in the same batch and cultured under consistent farming conditions, including feeding conditions, living environment, and culture period, and these differences also occur between genders. They may be caused by a variety of factors, such as genetics, health status, nutrient absorption capacity, and environmental conditions; however, the intestinal microbiota is likely to play an important role or be significantly affected. The red-claw crayfish gut harbors billions of bacteria that perform a variety of important activities for the host, which means it may be deeply involved in this size disparity. The intestinal microbiota plays key roles in various physiological activities such as digestion, nutrient absorption, energy regulation, metabolism, immunity, and disease prevention [6]. Screening and analyzing the microbial community’s structure, diversity, and abundance are essential means to elucidate their structural characteristics and further explore the possible influencing factors, which might contribute to developing high-quality practical nutritional strategies and optimal crayfish culture protocols [27].

Next-generation 16S rRNA gene sequencing technology has been applied to investigate the intestinal microbial communities in crustaceans. However, there is a lack of information on the intestinal microbial communities of *C. quadricarinatus* of various sizes and genders reared from the same larval batch in the same culture environment, as well as related gender-based differences. Given the importance of gut-associated microbial communities on animal growth, it is necessary to elucidate the gut microbial communities in *C. quadricarinatus* and the factors influencing them. Moreover, PICRUSt [28] was employed in this study to analyze the changes to microbial function based on the sequence abundance detected via 16S rRNA gene sequencing [3].

## 2. Materials and Methods

### 2.1. Experimental Animals

A total of 400 red-claw crayfishes were collected in three adjacent culture ponds in Sanjiang Farm, Haikou city, Hannan province, China, in November 2024. All the red-claw crayfishes were chosen from larvae of similar sizes (4.15 ± 0.34 cm; 7.43 ± 0.21 g) in the same batch. The three adjacent culture ponds were independent of each other, with the same floor area, rearing density, water source, and aquaculture setup. All the crayfishes were fed with commercial feed for 3 months under consistent farming conditions. (The environmental conditions are as follows: temperature, 28–32 °C, pH, 7.4–8.0, dissolved oxygen ≥ 6.0 mg/L, ammonia nitrogen ≤ 0.20 mg/L, nitrite ≤ 0.1 mg/L. The stocking density is originally 5000 crayfishes per 667 m^2^. The diet used in this study was a commercial feed purchased from Hainan Baiyang Feed Co., Ltd (Wenchang, China), with crude protein ≥ 38%, crude lipid ≥ 4.5%, crude fiber ≤ 5.0%, crude ash ≤ 15.0%. The crayfishes cultured in this study were fed twice per day: before 8:00 am and after 6:00 pm.) The experimental animals were randomly captured using cage traps in each culture pond. All crayfishes in the cages were collected and their body weights were measured. The crayfishes with the top 10% and bottom 10% daily weight gain were assigned to the high-body-weight group and low-body-weight group, respectively. In total, 30 larger-sized female (BF; 51.35 ± 5.66 g) and larger-sized male (BM; 67.23 ± 14.46 g) crayfishes were randomly chosen from the high-body-weight group, respectively. At the same time, 30 smaller-sized female (SF; 25.86 ± 4.99 g) and smaller-sized male (SM; 26.37 ± 4.69 g) crayfishes were randomly chosen from low-body-weight group (Supplementary Table S1). All crayfishes were dissected with sterilized and flamed surgical tools under sterile conditions. The gut was extracted and surface-sterilized via 75% ethanol three times before being rinsed three times in sterile phosphate-buffered saline (PBS). All gut samples (named GUBF, GUBM, GUSF, and GUSM) were ground in a sterile grinder (JXFSTPRP-24/32 (Shanghai Jingxin Industrial Development Co., Ltd., Shanghai, China) and stored at −80 °C immediately until use. Every five crayfish intestines from the same gender and size group were randomly mixed, subjected to DNA extraction, and considered as one sample. Each group included five replicates.

### 2.2. DNA Extraction and 16S rRNA Gene Sequencing

The gDNA of each sample was extracted using the commercial DNA Kit (DP302-02, Tiangen Biotech Co., Ltd., Beijing, China) according to the manufacturer’s instructions. The quality and quantity of DNA were assessed by agarose gel electrophoresis and Qubit (Thermo Fisher Scientific, Waltham, MA, USA). The V3-V4 region of the 16S rRNA genes was PCR-amplified from a DNA aliquot of the extracted gut sample using the forward primer 341F (5′-CCTAYGGGRBGCASCAG-3′) and the reverse primer 806R (5′-GGACTACNNGGGTATCTAAT-3′) with a barcode. PCR was performed as follows: 95 °C for 30 s, followed by 32 cycles at 95 °C for 10 s, 54 °C for 30 s, 72 °C for 45 s, and a final extension at 72 °C for 10 min in a 25 μL reaction mixture containing 12.5 μL of Phusion Hot start flex 2X Master Mix (New England Biolabs, Ipswich, MA, USA), 2.5 μL of each primer (Sangon Biotech Co., Ltd., Shanghai, China), and 50 ng of template DNA. The PCR products were detected by 2% agarose gel electrophoresis, and the samples with target electrophoresis bands were chosen for further experiments. PCR products with different barcodes were mixed in equal quantities and purified with AMPure XP beads (Beckman Coulter Genomics, Danvers, MA, USA) and quantified via Qubit (Thermo Fisher Scientific, Waltham, MA, USA). The purified PCR products were evaluated using an Agilent 2100 Bioanalyzer (Agilent Technologies, Santa Clara, CA, USA) and an Illumina library quantification kit (Kapa Biosciences, Woburn, MA, USA) following the manufacturer’s recommendations. The qualified library concentration should be above 2 nm. Each qualified sequencing library (with non-repetitive index sequences) was gradient-diluted, mixed in corresponding proportions according to the required sequencing volume, denatured into single strands with NaOH, and then sequenced on a machine. A NovaSeq 6000 sequencer (Illumina, Inc., San Diego, CA, USA) was used for 2 × 250 bp paired-end sequencing with the NovaSeq 6000 SP Reagent Kit (500 cycles, Illumina, Inc., San Diego, CA, USA).

### 2.3. Data Analysis 

After the sequencing primers were removed from de-multiplexed raw sequences using cutadapt (v1.9), the pair-ended clean reads were merged to clean tags using FLASH (v1.2.11) and assigned to each sample according to the unique barcodes. Low-quality reads (quality scores < 20), short reads (<100 bp), and reads containing more than 5% “N” records were trimmed by using the sliding-window algorithm method in fqtrim (v 0.94). Chimeric sequences were filtered via Vsearch software (v2.3.4). DADA2 (v1.16) was applied for denoising and generating amplicon sequence variants (ASVs). Sequence alignment of species annotation was performed by a QIIME2 plugin feature-classifier (v2024.2.0; https://qiime2.org/), and the alignment database was SILVA (https://www.arb-silva.de/; accessed on 24 February 2025) and NT-16S (https://www.ncbi.nlm.nih.gov/nucleotide/; accessed on 24 February 2025). Alpha and beta diversities were calculated using QIIME2 (v2024.2.0; https://qiime2.org/). Relative abundance was used in bacteria taxonomy. The Kruskal test was used to identify the differentially abundant phylum and genus, and significance was declared at *p* < 0.05. LDA effect size (LEfSe, LDA ≥ 3.0, *p* value < 0.05) analysis was performed via SegataLab/lefse. Other diagrams were implemented using the R package (v3.4.4).

### 2.4. Statistical Analysis

The amplicon sequence variants (ASVs) method for species classification was performed in this study, which typically employs a 100% identity threshold. The ASVs were used to annotate taxonomic information and further analysis. The rank abundance curve generated from amplicon sequence variants (ASVs) described species richness and evenness of a sample. Chao1, observed the species, and ace index estimated the community richness, evenness and species abundance, while, the Shannon index estimated the diversity in the GUBF, GUBM, GUSF, and GUSM groups. Beta diversity refers to species differences between communities (samples) in different environments. In this case, the unweighted-unifrac algorithm was employed to compare the distance between samples and principal coordinates analysis (PCoA), non-metric multidimensional scaling (NMDS), and analysis of similarities (ANOSIM) were conducted to evaluate the beta diversity between the various groups (the GUBF, GUBM, GUSF, and GUSM groups). A *p* value of 0.05 is used as a threshold for statistical significance. Based on the Kruskal–Wallis rank sum test and Wilcoxon rank sum test, a linear discriminant analysis (LDA) effect size (LEfSe) analysis was performed with the parameters of LDA > 3.0 and *p* < 0.05. PICRUSt2 (Phylogenetic Investigation of Communities by Reconstruction of Unobserved States, v2.4.1; https://github.com/picrust/picrust2) analysis was conducted based on the KEGG database (KEGG pathway database, Level 3, http://www.genome.jp/kegg/pathway.html, accessed on 24 February 2025) for biological metabolic pathway analysis. Based on the relative abundance tables of each classification level in the database, the subsequent analyses can be performed using officially recommended STAMP differential analysis. The BugBase (v1.0; developed by the Knight Lab, University of California, San Diego, CA, USA) analysis was performed based on Rob Knight Lab’s instructions [29] to predict the phenotypes of the microbiome samples.

## 3. Results 

### 3.1. Alpha and Beta Diversity Analysis

In total, 1,680,282 intestinal microbial 16S rRNA gene raw reads were assembled via FLASH, and 1,482,226 trimmed tags were obtained from the gut content of the GUBF, GUBM, GUSF, and GUSM groups, respectively (Table 1). The average number of trimmed tags per sample was 74,111, indicating that the sequencing depth and sampling amount were sufficient to cover the majority of the microbial community. All sequences were clustered into ASVs according to a 100% identity threshold, and a total of 6840 ASVs and an average of 342 ASVs per sample were obtained for further analysis (Table 1). Meanwhile, each phylum to genus involved across all samples was identified (Table 1).

**Figure 1:**
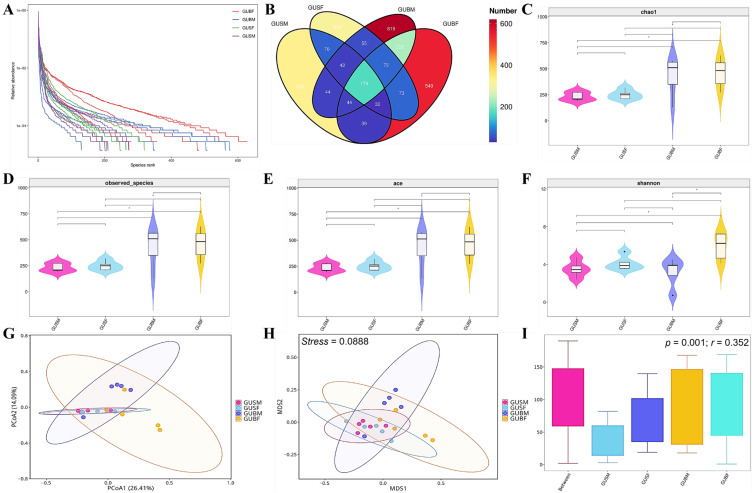
The alpha and beta diversities among the GUBF, GUBM, GUSF and GUSM groups. (**A**) The rank abundance curve showing the species richness and evenness of gut microbiota in all samples; (**B**) Venn diagram plot showing the unique and shared ASVs among the GUBF, GUBM, GUSF, and GUSM groups; ((**C**), Chao1), ((**D**), observed species), ((**E**), ace), the alpha diversity index (Chao1, observed species, and ace) showing the community richness of the intestinal microbiota in the GUBF, GUBM, GUSF, and GUSM groups; (**F**) Shannon index showing the community diversity of the intestinal microbiota in the GUBF, GUBM, GUSF, and GUSM groups; ((**G**), PCoA), ((**H**), NMDS), ((**I**), ANOSIM), PCoA, NMDS, and ANOSIM analysis based on unweighted-unifrac distance showing the gut microbiota clustering in a size- and gender-dependent manner.

Furthermore, the dynamics of alpha diversity were further studied (Figure 1). The rank abundance curve revealed that the species richness and evenness were highest in the larger-sized female crayfish (GUBF), followed by the larger-sized male crayfish (GUBM), the smaller-sized female crayfish (GUSF), and the smaller-sized male crayfish (GUSM, Figure 1A). The Venn diagram plot presents the ASV distribution in the GUBF, GUBM, GUSF, and GUSM groups. We identified that there were 1212, 1281, 827, and 772 ASVs in each individual group; 174 ASVs shared by all groups; and 549, 619, 310, and 329 ASVs were unique to one group (Figure 1B). The chao1, observed species, and ace indexes were employed, suggesting that the community richness in different sample groups was remarkably decreased in the following order: GUBF, GUBM, GUSF, and GUSM. Specifically, significant differences were found between the GUBF and GUSF groups, as well as between the GUBF and GUSM groups (Figure 1C–E). On the other hand, the Shannon index decreased in the order of GUBF, GUSF, GUSM, and GUBM, indicating that the community diversity was significantly highest in the GUBF group, but lowest in the GUBM group (Figure 1F).

For beta diversity analysis, PCoA and NMDS, as well as the ANOSIM analysis based on unweighted-unifrac distance, revealed that gut microbiota clustering occurred in a size- and gender-dependent manner (Figure 1G–I). The clustering of the intestinal microbiota in the GUSM and GUSF groups almost completely overlapped, and both were nearly entirely covered within the clustering range of either GUBF or GUBM. Meanwhile, the clustering of the GUBF and GUBM groups exhibited distinct tendencies, suggesting notable differences in the diversity and abundance between the GUBF and GUBM groups, possibly due to size and gender.

### 3.2. Taxonomic Composition and Dynamics of Gut Microbiota

**Figure 2:**
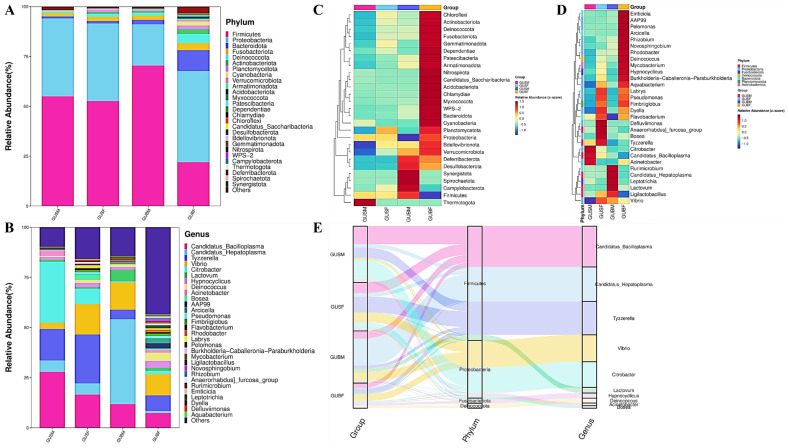
The relative abundances of the dominant microbiota at phylum and genus levels in the GUBF, GUBM, GUSF, and GUSM groups. (**A**) The elative abundances of the dominant microbiota at the phyla level; (**B**) the relative abundances of the dominant microbiota at the genus level; (**C**) a heatmap clustering plot showing the abundant phyla in the GUBF, GUBM, GUSF, and GUSM groups; (**D**) a heatmap clustering plot showing the abundant genera in the GUBF, GUBM, GUSF, and GUSM groups; (**E**) Sankey plot showing the species annotation information, corresponding relationships, and proportions of the dominant phyla, genera, and experimental groups.

A total of 28 phyla were identified in all samples. The dominant phyla (abundance proportion > 1%) are listed in Table 2. *Firmicutes* was the first dominant phylum among individuals in the GUBM (70.62%), GUSM (55.10%), and GUSF (52.79%) groups (Table 2). However, *Proteobacteria* was the most abundant phylum in the GUBF group (70.62%; Table 2). *Firmicutes*, *Proteobacteria*, and *Fusobacteriota* were stable as the core dominant phyla in all four experimental groups. *Firmicutes*, *Proteobacteria*, *Bacteroidota*, *Fusobacteriota*, and *Deinococcota* were dominant microbiota in the GUBM, GUSF, and GUBF groups, though this did not hold true for the GUSM group, which only had *Firmicutes*, *Proteobacteria*, *Fusobacteriota*, and an unclassified phylum (Table 2; Figure 2A). Bacteroidota and *Fusobacteriota* were abundant in the GUBM group compared with the GUSM group (Table 2, Figure 2A). In comparison of the GUBF and GUSF groups, it was found that *Actinobacteriota* and *Cyanobacteria*, as well as an unclassified phylum, were dominant in the GUBF group (Table 2; Figure 2A). There were many more microbial phyla abundant in the gut microbiota of female individuals, regardless of whether they had a larger or smaller body size (Table 2; Figure 2A).

On the other hand, 673 genera were identified in all the samples, with 25 core genera (abundance proportion > 1%; Table 2). *Citrobacter*, *Candidatus_Hepatoplasma*, *Tyzzerella*, and *Vibrio* were the first dominant genera among individuals in the GUSM (30.43%), GUBM (42.28%), GUSF (24.29%), and GUBF (10.50%) groups (Table 2; Figure 2B). The genera of *Vibrio*, *Tyzzerella*, *Candidatus_Bacilloplasma*, *Candidatus_Hepatoplasma*, and *Mycoplasmataceae_unclassified* were found to be much abundant in all samples, indicating that they are the core genera in the gut microbiota of red-claw crayfishes (Table 2; Figure 2B). Moreover, *Rhodobacteraceae_unclassified*, *Candidatus_Hepatincola_unclassified*, *Pseudomonas*, and *Fimbriiglobus* were abundant in both the GUSF and GUBF groups, but not in male samples, while *Citrobacter* showed relatively high abundance in the smaller-sized groups, like the GUSM (30.43%) and GUSF (7.77%) groups. *Lactovum* was abundant in the larger-sized groups, including GUBM (5.77%) and GUBF (1.46%). The heatmap included in this study presents the significantly enriched phyla and genera in the different groups, clearly suggesting that the enriched phyla and genera were notably different among the GUBF, GUBM, GUSF, and GUSM groups (Figure 2C,D). In addition, the relationship of the significantly enriched phyla and genera is exhibited in Figure 2E.

### 3.3. Significantly Different Phyla and Genera Between Different Groups

In this study, the abundance of nine phyla and 106 genera in the intestinal microbiota of all crayfish groups were identified to be significantly different in abundance over all four groups (Supplementary Table S2). Pairwise comparisons revealed that the significantly regulated phyla and genera varied (Table 3). The abundance of seven, six, two, and four phyla were notably modulated between the GUBF and GUBM groups, the GUBF and GUSF groups, the GUBM and GUSM groups, and the GUSF and GUSM groups, respectively (Table 3). A comparison with the GUBM group indicated that the abundance of *Acidobacteriota*, *Myxococcota*, *Dependentiae*, *Bacteroidota*, and *Planctomycetota* was significantly up-regulated in the GUBF group, but the abundance of *Campylobacterota* and *Firmicutes* notably declined (Table 3). Compared with the GUSF group’s abundance of *Acidobacteriota*, *Myxococcota*, *Bacteroidota*, and *Cyanobacteria*, the abundance of *Campylobacterota* was significantly increased in the GUBF group, while the abundance of *Firmicutes* was decreased in the GUBF group. The abundance of *Chloroflexi* and *Verrucomicrobiota* in the GUBM group was significantly up-regulated, and the abundance of *Dependentiae*, *Actinobacteriota*, *Chloroflexi*, and *Planctomycetota* in the GUSF group was remarkably up-modulated compared to the GUSM group (Table 3).

At the genus level, 55, 55, 48, and 4 differently regulated genera, in terms of abundance, were found in the comparison between the GUBF and GUBM groups, the GUBF and GUSF groups, the GUBM and GUSM groups, and the GUSF and GUSM groups, respectively (Table 3). Through the comparison of the genus abundance between the GUBF and GUBM groups, 39 and 16 genera were significantly increased and decreased, respectively (Table 3). In the comparison of the GUBF and GUSF groups, the abundance of 52 genera was significantly up-modulated and that of 3 genera was down-modulated. Similarly, in our comparison of the GUBM and GUSM groups, 47 genera were significantly increased, and 1 genus was significantly decreased. All four genera in the GUSF group were significantly up-regulated compared with the GUSM group (Table 3).

### 3.4. Variance Analysis

**Figure 3:**
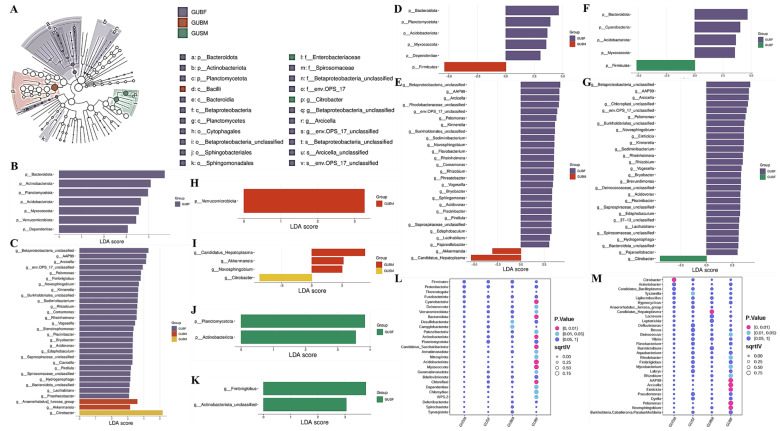
LEfSe analysis displaying the differences in intestinal microbiota. (**A**) A cladogram showing differentially abundant taxa of the intestinal microbiota among the GUBF, GUBM, GUSF, and GUSM groups; (**B**, Phyla), (**C**, Genus), differential abundance of the phyla and genera among the four experimental groups (LDA > 3, *p* value < 0.05); (**D**,**E**) differential abundance of the phyla and genera between the GUBF and GUBM groups; (**F**,**G**) differential abundance of the phyla and genera between the GUBF and GUSF groups; (**H**,**I**) differential abundance of the phyla and genera between the GUBM and GUSM groups; (**J**,**K**) differential abundance of the phyla and genera between the GUSF and GUSM groups; (**F**,**G**) differential abundance of the phyla and genera between the GUBF and GUSF groups; (**L**,**M**) taxonomy indicator bubble plot showing the potential phyla and genera biomarkers among the GUBF, GUBM, GUSF, and GUSM groups.

LEfSe analysis was conducted to screen for a potential biomarker in the gut microbiota (Figure 3). A cladogram plot revealed the significantly enriched microbial communities at different levels of classification in the GUBF, GUBM, and GUSM groups (see Figure 3A; no microbial taxa with significance were identified in the GUSF group via LEfSe LDA > 3, *p* < 0.05). In total, 22 taxa were found via the cladogram plot, including 19 taxa in the GUBF group, 1 taxon in the GUBM group, and 2 taxa in the GUSM group (Figure 3A). Furthermore, comparison among all four groups suggested that seven phyla and 30 genera were significantly enriched in abundance, including seven phyla and 27 taxa in the GUBF group, two taxa in the GUBM group, and one taxon in the GUSM group (Figure 3B,C). Pairwise comparisons were performed based on body size and gender. There were six, five, one, and two phyla and 27, 28, 4, and 2 genera identified to be significantly different in abundance in the comparison of GUBF and GUBM, GUBF and GUSF, GUBM and GUSM, and GUSF and GUSM, respectively. *Bacteroidota*, *Acidobacteriota*, and *Myxococcota* were significantly enriched in the GUBF group, regardless of whether we were comparing the GUBM or GUSF group, and *Firmicutes* was found to be enriched in both the GUBM and GUSF groups in a comparison with the GUBF group (Figure 3D,F). Between the GUBM and GUSM groups, phylum *Verrucomicrobiota* and genera *Candidatus_Hepatoplasma*, *Akkermansia*, and *Novosphingobium* were all significantly enriched in the GUBM group, and only Citrobacter was notably enriched in the GUSM group (Figure 3H,I). The other phyla and genera with significant differences in abundance are exhibited in Figure 3B–K.

On the other hand, indicator species analysis was employed to search for biomarkers with specific indicative significance. The results derived from our comparison among the four groups showed no potential biomarkers in the GUSM and GUSF groups. Only two phyla, *Campylobacterota* and *Desulfobacterota*, were significantly enriched in the GUBM group (*p* < 0.05; Figure 3L). Plenty of phyla were significantly enriched in the GUBF group, including *Cyanobacteria*, *Bacteroidota*, *Actinobacteriota*, *Candidatus_Saccharibacteria*, *Acidobacteriota*, *Myxococcota*, and *Chloroflexi* (*p* < 0.01; Figure 3L). At the genus level, *Citrobacter* (*p* < 0.01)*, Tyzzerella* (*p* < 0.05), and *Candidatus_Hepatoplasma* (*p* < 0.01) were significantly enriched in the GUSM, GUSF, and GUBM groups, respectively (Figure 3M). In the GUBF group, *AAP99*, *Arcicella*, *Emticicia*, *Pelomonas*, and *Novosphingobium* were significantly enriched (*p* < 0.01; Figure 3M).

In general, both the LEfSe analysis and indicator species analysis showed that the phylum Citrobacter in the GUSM group (Figure 3I) and the phyla *Bacteroidota*, *Actinobacteriota*, *Acidobacteriota*, and *Myxococcota* and genera *AAP99*, *Arcicella*, *Pelomonas*, *Novosphingobium*, and *Rhizobium* in the GUBF group were significantly enriched in the intestinal microbiota and could be potential biomarkers for their respective groups (Figure 3C,L,M). Moreover, the Manhattan plot included in this study shows the significantly enriched or depleted intestinal microbial taxa (phylum) via a pairwise comparison pattern, which may offer additional support of the aforementioned outcomes (Supplementary Figure S1).

### 3.5. Correlation Analysis

**Figure 4:**
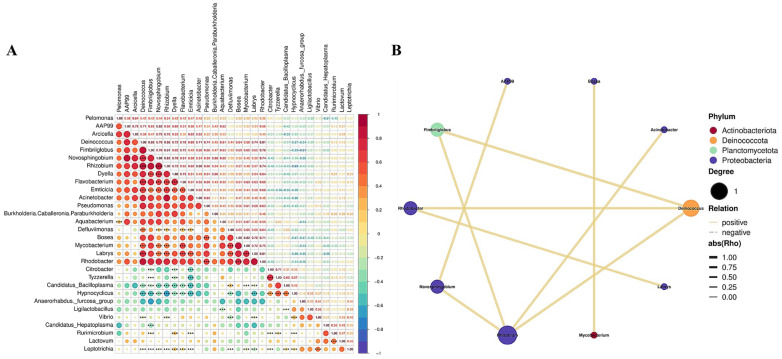
The correlation heatmap and network plot displaying the correlations between the different genera and phyla. (**A**) A correlation heatmap plot showing the significant correlations between the top 30 dominant genera in the experimental groups. Statistical significance is marked as follows: *** for *p* < 0.001. (**B**) A network diagram showing the correlation between the different dominant genera and the connections between nodes displaying the correlations between two genera with a correlation coefficient |*rho*| > 0.8.

The correlation heatmap illustrates the significance of correlations among different genera (Figure 4A). The genus *Rhizobium* had positive correlations with the genera *Flavobacterium*, *Emticicia*, *Mycobacterium*, *Labrys*, and *Defluviimonas* (*p* < 0.001) but negative correlations with *Candidatus_Bacilloplasma*, *Hypnocyclicus*, *Vibrio*, and *Leptotrichia* (Figure 4A). *Deinococcus* had positive correlations with the genera *Novosphingobium*, *Rhizobium*, *Flavobacterium*, *Emticicia*, *Defluviimonas*, *Mycobacterium*, and *Labrys*, but negative correlations with *Candidatus_Bacilloplasma*, *Hypnocyclicus*, *Vibrio*, and *Leptotrichia* (Figure 4A). More correlations and significant differences between the different dominant genera are listed in Figure 4A.

On the other hand, correlation analysis revealed the relationships between various phyla and genera. The network plot revealed the correlations between the dominant genera in the intestinal microbiota of all experimental groups and the respective groups each phylum belongs to (Figure 4B). *Rhizobium*, *Deinococcus*, *Fimbriiglobus*, *Rhodobacter*, *Novosphingobium*, *Acinetobacter*, *Mycobacterium*, *Labrys*, *AAP99*, and *Bosea* were all in a positive relationship with each other (|*rho*| > 0.8; Figure 4B). Moreover, *Rhizobium* and *Deinococcus* had a positive interaction with the three other dominant genera, indicating a central position within the intestinal microbiota (Figure 4B). *Fimbriiglobus*, *Rhodobacter*, and *Novosphingobium* shared a positive relationship with two other dominant genera, suggesting that they are important components of the intestinal microbiota (Figure 4B).

### 3.6. KEGG PICRUSt2 Analysis 

**Figure 5:**
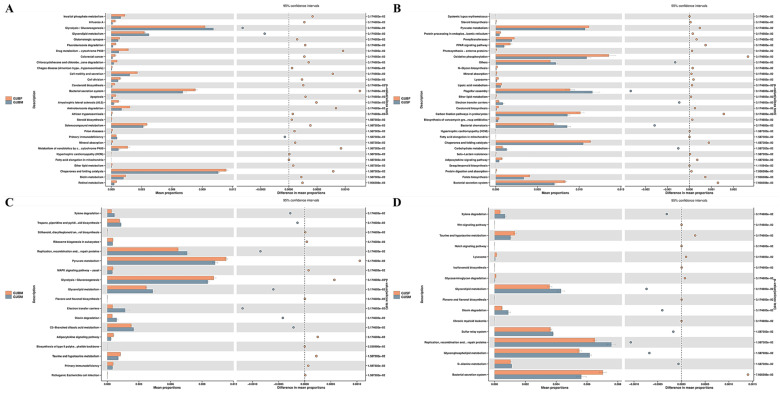
STAMP analysis displaying the intestinal microbiota KEGG functional classification chart between the different groups. (**A**) The significantly regulated pathways between the GUBF and GUBM groups; (**B**) the significantly regulated pathways between the GUBF and GUSF groups; (**C**) the significantly regulated pathways between the GUBM and GUSM groups; (**D**) the significantly regulated pathways between the GUSF and GUSM groups.

In this study, PICRUSt2 was employed to predict functional abundance based on marker gene sequences based on the KEGG database (level 3). The first 30 significantly enriched pathways (*p* < 0.05, if the number of pathways was more than 30), with significance based on pairwise comparison, are listed in Figure 5. The comparison between the GUBF and GUBM groups identified 27 and 3 pathways in the GUBF and GUBM groups, respectively. The top five significantly enriched pathways in the GUBF group were as follows: chaperones and folding catalysts, bacterial secretion system, selenocompound metabolism, cell motility and secretion, and aminobenzoate degradation (Figure 5A). Meanwhile, glycolysis/gluconeogenesis, glycerolipid metabolism, and primary immunodeficiency were significantly enriched in the GUBM group (Figure 5A). Additionally, 25 and 5 pathways were significantly enriched in the GUBF and GUSF groups, including oxidative phosphorylation, pyruvate metabolism, chaperones and folding catalysts, carbon fixation pathways in prokaryotes, and bacterial secretion system in the GUBF group, while flagellar assembly, bacterial chemotaxis, carbohydrate metabolism, and electron transfer carriers were the specific pathways enriched in the GUSF group (Figure 5B). In our comparative analysis, 11 and 7 pathways were identified to be significantly enriched in the GUBM and GUSM groups, respectively. Pyruvate metabolism, glycolysis/gluconeogenesis, taurine and hypotaurine metabolism, adipocytokine signaling pathway, and primary immunodeficiency were the top five pathways enriched in the GUBM group, and replication, recombination, and repair proteins; glycerolipid metabolism; C5-branched dibasic acid metabolism; electron transfer carriers; and tropane, piperidine, and pyridine alkaloid biosynthesis were the top five pathways enriched in the GUSM group (Figure 5C). Comparison between the GUSF and GUSM groups identified that nine pathways were enriched in the GUSF group, including bacterial secretion system, taurine and hypotaurine metabolism, and glycosaminoglycan degradation, and seven pathways were enriched in the GUSM group, including replication, recombination, and repair proteins; glycerophospholipid metabolism; and glycerolipid metabolism (Figure 5D).

In addition, the comprehensive pairwise comparison analysis of the GUBF and GUBM groups and the GUSF and GUSM groups indicated that the following pathways were significantly up- and down-regulated in female individuals regardless of body size: bacterial secretion system, isoflavonoid biosynthesis, and glycerolipid metabolism (Figure 5A,D; Supplementary Table S3). Another combined pairwise comparison of the GUBF and GUSF groups and the GUBM and GUSM groups showed that the adipocytokine signaling pathway, pyruvate metabolism, and electron transfer carriers were significantly up- and down-regulated in larger individuals regardless of gender (Figure 5B,C; Supplementary Table S3).

### 3.7. BugBase Analysis 

**Figure 6:**
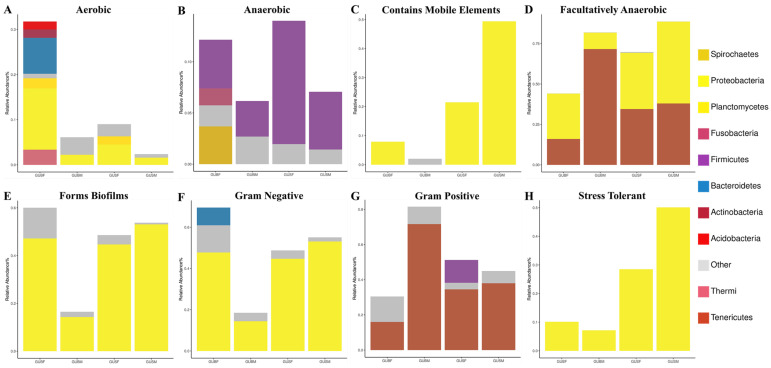
A stacked bar chart displaying the relative abundance of phyla with different phenotypes across the GUBF, GUBM, GUSF and GUSM groups. (**A**) Aerobic; (**B**) anaerobic; (**C**) contains mobile elements; (**D**) facultatively anaerobic; (**E**) forms biofilms; (**F**) Gram-negative; (**G**) Gram-positive; (**H**) stress-tolerant.

With the BugBase tool, the phenotypes of intestinal microbial samples can be predicted as nine potential phenotypes: aerobic, anaerobic, contains mobile elements, facultatively anaerobic, forms biofilms, Gram-negative, Gram-positive, potentially pathogenic, and stress-tolerant. In this study, eight potential phenotypes, namely, aerobic, anaerobic, contains mobile elements, facultatively anaerobic, forms biofilms, Gram-negative, Gram-positive, and stress-tolerant, were identified in the intestinal microbial communities, and included 10 dominant phyla (Figure 6). The experimental groups with the highest cumulative relative intestinal microbiota abundance in each phenotype are as follows: the GUBF group in the aerobic, forms biofilms, and Gram-negative phenotypes (Figure 6A,E,F); the GUBM group in the Gram-positive phenotype (Figure 6D); the GUSF group in the anaerobic phenotype (Figure 6B); and the GUSM group in contains the mobile elements, facultatively anaerobic, and stress-tolerant phenotypes (Figure 6C,D,H). Conversely, the lowest cumulative relative intestinal microbiome abundance for the GUBF, GUBM, and GUSM groups appeared in the facultatively anaerobic and Gram-positive phenotypes (Figure 6D,G); the anaerobic, contains mobile elements, forms biofilms, Gram-negative, and stress-tolerant phenotypes (Figure 6B,C,E,F,H), and the aerobic phenotype (see Figure 6A; there was no phenotype in which GUSF had the lowest cumulative relative abundance), respectively.

Moreover, the compositional distribution of the intestinal microbiota in the experimental groups with the highest or lowest cumulative abundance varies in each phenotypic category (Figure 6). For example, the top five in terms of the compositional distribution of the intestinal flora in the GUBF group in the aerobic phenotype were *Proteobacteria*, *Bacteroidetes*, *Fusobacteria*, *Planctomycetes*, and *Actinobacteria* (Figure 6A); however, *Proteobacteria* and *Other* were found for the forms biofilms phenotype (Figure 6E), and *Proteobacteria*, *Other*, and *Bacteroidetes* were found for the Gram-negative phenotype (Figure 6F). This might be one of the possible reasons for the various size- and gender-based differences in red-claw crayfish.

## 4. Discussion 

The present study aimed to first characterize the composition of the gut microbiota of *C. quadricarinatus* with various sizes and genders reared in the same culture environment and period and from the same batch, with all crayfish having similar body weights. Then we aimed to evaluate the impact of body size and gender on the intestinal microbiota communities. Residential microbes have performed metabolic functions in animals for at least 500 million years, and the extensive congruent phylogenies of animal hosts and their microbiota, involving both individual organisms and whole microbial populations, suggest the existence of specific selection based on co-adaptation [22]. The intestinal microbiota has been proven to play vital roles in aquatic animals’ digestion and absorption, metabolism, development, and immunity [2,30]. The composition and distribution of intestinal microbiota are synergistically shaped by the internal and external factors of the host [27,30], namely, genetic characteristics, gender, and developmental stage as internal factors, as well as geographic location, breeding patterns, and dietary conditions as the main external factors. Numerous studies have demonstrated that the intestinal microbiota is influenced by single internal or external factors, or by the synergistic interaction of both [30,31,32,33].

However, a common phenomenon in aquaculture production is that seedlings of similar quality, derived from the same batch of parents and reared in a consistent culture environment for a certain period, exhibit inconsistent sizes. A study on the cultured large yellow croaker (*Pseudosciaena crocea*) revealed that the core intestinal microbiota differs among individuals with varying growth rates [34], yet the impact of gender differences on the intestinal microbiota remains unexplored. Given that sex bias is prominently manifested in multiple physiological aspects of aquatic animals, it may potentially exert a significant influence on the intestinal microbiota. Jiang reported that the intestinal microbiota patterns of Chinese mitten crabs (*E. sinensis*) vary between genders [30]. Nevertheless, little is known about differences in the intestinal microbiota among red-claw crayfish of the same gender but with varying body sizes and reared in the same culture environment. This could be attributed to multiple factors; however, as an important part in nutrient digestion and absorption in aquatic animals, the intestinal microbiota may have a significant influence. The changing of the dominant phyla from embryonic stages to post-larval stages in *M. rosenbergii* was attributed to promoting prawn growth and physiological health [35]. Moreover, bacterial communities may enhance the production of cellulose-degrading enzymes and help their hosts adapt optimally to their environment by regulating host metabolism [35]. On the other hand, diet can also impact the host intestinal microbial community and affect host growth and health. Nutrient competition is the main mechanism by which intestinal microbiota inhibits Salmonella colonization, thereby extending mice survival [36]. The composition of the intestinal microbial community is crucial for enhancing digestion and absorption efficiency, as well as improving growth and immune response in shrimp [35].

In this study, the structure and distribution of intestinal microbiota, the relationship between the core microbiota, and the body size and gender specificity of intestinal microbiota were comprehensively assessed. Alpha and beta diversity analysis of all four groups indicated that the species richness, evenness, diversity, and abundance exhibited body size (weight) and gender patterns. When the gender is the same, the above-mentioned indices in the larger-sized group were significantly higher than those in the smaller-sized group, whereas among the groups with similar individual sizes, these indices were relatively more abundant in the female group compared to the male group (Figure 1). It is reasonable to assume that larger crayfish may hold a greater advantage during predation, thereby securing more adequate food resources. However, processing and utilizing such abundant food likely requires intestinal microbiota with higher diversity and abundance to facilitate efficient digestion and absorption. In turn, improved nutrient intake can promote molting and growth, ultimately resulting in larger body sizes. Conversely, larger female individuals possess more internal space to accommodate hepatopancreas and ovarian development. Ovarian growth, in particular, stimulates the production of more sex hormones and demands substantial quantities of diverse nutrients, which may depend on intestinal microbiota with enhanced diversity and abundance to support the uptake of sufficient and varied nutrients for hormone synthesis or consumption. As several studies reported, the sex differences in gut microbiota do not appear until puberty, indicating that the role of sex hormones in shaping the gut microbiota composition is supported [37]. Sexual dimorphism is regulated by sex hormones, which interact bidirectionally with the intestinal microbiota. Sex hormones influence the microbiota by modulating intestinal barrier permeability and integrity, as well as by affecting sex hormone receptors, β-glucuronidase, bile acid, and intestinal immunity [38]. Conversely, the intestinal microbiota impacts the secretion of sex hormones, such as androgen. Testosterone and ovaries are the primary sources of male and female androgen, respectively, and the intestinal microbiota plays a key role in regulating androgen metabolism in the gut [38]. The microbiota compositions of male mice deviated, suggesting that male sex hormones may play an important role in the sex differences in gut microbiota in mice [37]. Moreover, the interaction between estrogen and gut microbiota has been documented. Specifically, in males and postmenopausal females, but not in premenopausal females, there is a significant association between total urinary estrogen levels and the richness and alpha diversity of intestinal microbiota [37,38,39]. Furthermore, soy isoflavones, which have metabolites structurally similar to estrogen, can significantly alter the intestinal microbial community in postmenopausal women by increasing the concentration of Bifidobacterium while suppressing unclassified *Clostridiaceae* [40]. Interestingly, the isoflavonoid biosynthesis pathway was significantly up-regulated in the female individuals, regardless of body sizes, which may suggest a potential interaction between the sex hormone and gut microbiota. Additionally, after restricting oviposition in honeybees, the weight of the queen bee’s ovaries was significantly decreased, gut microbiota diversity declined, and the core gut microbiota changed [41], suggesting that an interaction exists between the ovaries and gut microbiota. Moreover, the content of phosphatidylcholine, phosphoethanolamine (*p* < 0.001), and 1-acyl-sn-glycero-3-phosphocholine was significantly increased in the ovaries, indicating that the glycerophospholipid metabolism was up-regulated [41]. This is consistent with our observations: the pathway glycerolipid metabolism were significantly down-regulated in the female individuals, in which the ovaries developed normally, regardless of body sizes. The detailed interaction processes between ovary and gut flora require further exploration and verification in crayfish. Moreover, regardless of body size and gender, *Firmicutes*, *Proteobacteria*, and *Fusobacteriota* were stable as the main dominant phyla in all four experimental groups. *Bacteroidota* was the dominant phylum in the GUSM, GUBM, and GUBF groups, and *Deinococcota* was the dominant phylum in the GUSF, GUBM, and GUBF groups (Table 2). These results were generally consistent with the core microbiota of *C. quadricarinatus* in Jiaxing, Zhejiang, and the red swamp crayfish (*P. clarkii*) in Jingzhou Hubei, Yangzhou, and Xuyi Zhejiang [25]. The difference might be due to the geographic location, climate conditions, and temperature (all crayfishes used in this study were captured from the subtropical region in China, Hainan). *Proteobacteria*, highly diverse in terms of physiology, morphology, and genetics, are widespread in gut microbiotas of aquatic invertebrates and are often a dominant component of this community in Crustacea [42]. *Firmicutes* and *Bacteroidetes* have been reported to exist in mutually promoting symbiotic relationships between them [15], and have been reported to be the dominant phylum in many marine and freshwater crustaceans, such as freshwater shrimp (*M. nipponensis*), red swamp crayfish (*P. clarkii*), seawater shrimp (*L. vannamei* and *Penaeus monodon*), Chinese mitten crabs (*E. sinensis*), and mud crabs (*S. paramamosain*) [30,35,43,44,45]. Both of them have been proven to play a key role in intestinal homeostasis, and the stable core microbiota is beneficial to the intestinal homeostasis of the crayfish stock [30]. Moreover, microbial species belonging to *Bacteroidetes* can produce carbohydrate metabolism-related enzymes to promote food digestion [35,46], and are capable of producing propionate, which has the potential to alleviate colitis and enhance intestinal barrier function while reducing inflammation [47]. *Firmicutes* (*Proteobacteria* as well) is necessary for some physiological and biochemical functions of the intestine of crustacean species [44]. *Firmicutes* species produce a series of digestive enzymes to promote host digestion and nutrient absorption, potentially metabolize dietary plant polysaccharides, and are related to fatty acid absorption and metabolism [43,44,48,49,50,51,52,53]. Both of them stimulate the digestion and nutrient absorption of the host and produce energy for the host [35,43,44]. Furthermore, in the gut flora of juvenile *E. sinensis* fed with only freshwater snails, both freshwater snails and waterweed plants and only waterweed plants, the proportion of *Firmicutes* decreased from 16.87%, to 12.47%, to 5.07%, while the proportion of *Bacteroidetes* increased from 20.71%, to 31.20%, to 45.62% [44], indicating that the proportion of *Firmicutes* or *Bacteroidetes* declined or increased with the rise in the plant- or animal-originate sources in the diet [44]. In this study, the proportion of *Firmicutes* is 55.10%, 52.79%, 70.62%, and 22.10% in the GUSM, GUSF, GUBM, and GUBF groups, respectively (Table 2). At the same time, the proportion of *Bacteroidetes* is 0.95%, 1.23%, 2.24%, and 10.52% in the GUSM, GUSF, GUBM, and GUBF groups, respectively. These results suggested that the individuals in the GUSM, GUSF, and GUBM groups and animals in the GUBF group differed in consuming animal-sourced food, whereas animals in the GUBF group may have different preferences regarding plant-based or animal-based sources of raw materials in feed, and their rates of feed utilization may also vary. To confirm or deny this, additional detailed diet studies will be necessary in the future.

At the genus level, *Vibrio*, *Tyzzerella*, *Candidatus_Bacilloplasma*, *Candidatus_Hepatoplasma*, and *Mycoplasmataceae_unclassified* were found to be highly abundant in all the samples, suggesting that they are the core genera in the gut microbiota of red-claw crayfishes (Table 2; Figure 2B). Species from the *Vibrio* genus are indigenous to aquatic environments and widely distributed across aquaculture systems [54]. Though some *Vibrio* species and strains are pathogenic and infectious, leading to the disease “*Vibriosis*” [54], a large number of *Vibrio* species are non-pathogenic and commonly present in healthy farmed aquatic organisms, which means they may play a key role as part of the whole bacterial community structure [52,55]. The Vibrio genus commonly represents 30% of the total sequence of the gut microbiota of freshwater crayfish [54,55]. In this study, the abundance of the *Vibrio* genus showed no significant differences among all experimental groups, and no significance was found in all the pairwise comparisons (Table 3).

In addition, *Citrobacter* and *Lactovum* were identified to be significantly more abundant in smaller and larger individuals, respectively. The *Citrobacter* genus was discovered to be abundant in both the GUSM (30.43%) and GUSF (7.77%) groups, being significantly abundant in these groups compared to the GUBM (0.58%) and GUBF (1.75%) groups, indicating that *Citrobacteris* notably dominant in smaller-sized individuals. As a common bacterium in the intestines of red swamp crayfish (*P. clarkii*) and freshwater shrimp (*M. nipponensis*) [56,57], *Citrobacter* species are cellulose-degrading bacteria abundantly found in the intestines of herbivorous and omnivorous aquatic organisms, and are able to metabolize a remarkable variety of substrates, including fibrins in diets [56,58]. Given that daily diet greatly affects the microbiome of the digestive tract, the modulated composition and diversity of the gut microbiome affect host health status and induces alterations of host physiology, possibly including nutrient absorption, generation of tissue, and morphogenesis [56,59]. The abundance of the *Citrobacter* genus in the gut flora of the smaller animal individuals may indicate that they have a higher proportion of plant-derived nutrients food in their diet, which may delay their growth rate. On the other hand, Zhang et al. (2020) reported that a high-fat diet increased the number of *Citrobacter* spp. in the gut of Nile tilapia and increased high-fat diet-induced lipid accumulation in mesenteric adipose tissue, accompanied by increased triglyceride ab-sorption efficiency, triglyceride re-esterification, and increased intestinal permeability [60]. However, this still needs to be tested in crustaceans. As this study revealed, the genus *Lactovum* was abundant in the larger-sized groups, including GUBM (5.77%) and GUBF (1.46%). *Lactovum* genus chemo-organotrophic and characterized by an aerotolerant anaerobic metabolism that ferments glucose to lactate. Moreover, bacteria of the genus *Lactovum* exhibit a mixed fermentation metabolic mode to produce varying amounts of lactic acid, ethanol, formic acid, and acetic acid, depending on the substrates utilized [61]. The type species, *Lactovum miscens*, is isolated from the acidic forest soil [55,58], which possibly indicated that the enriched genus *Lactovum* in the larger crayfishes originated from pond sediments. Further, *Lactovum* is usually enriched and isolated on N-acetylglucosamine [62], a basic component of chitin, which is a component of arthropod biomass [63]. The significant abundance of the *Lactovum* genus in the larger crayfishes revealed that the proportion of digestion and absorption of various carbohydrates and metabolism of the sugar derivative from chitin was notably elevated in the larger individual animals, regardless of gender. However, further research needs to be conducted to confirm the above hypothesis.

On the other hand, the comparisons with the GUBF and GUBM groups and the GUSF and GUSM groups showed other changes between the male and female individuals. The pairwise comparisons with the GUBM group indicated that five and two phyla were significantly abundant or insufficient in the GUBM groups, respectively, and four genera were notably abundant in the GUSF groups compared with the GUSM group (Table 3). *Dependentiae* and *Planctomycetota* were both significantly abundant in female individuals, irrespective of body size. These results may suggest that there are more diverse and abundant microbiota in the gut of female crayfishes. In addition, 39 significantly abundant genera and 16 significantly insufficient genera were identified in comparison between the GUBF and GUBM groups, but only 4 significantly abundant genera were recognized between the GUSF and GUSM groups, indicating that the impact of gender on the gut microbiota was more pronounced and diverse in larger individual animals. The composition and abundance of core microbiotas can be greatly affected by gender. Since more diverse gut communities exert greater protective effects on the host, the homeostatic balance of the intestinal microbiota in the GUBF group individuals might be much stronger in the other groups.

The KEGG pathway predicted by PICRUSt2 showed that differences in body size and gender clearly impacted the metabolic pathways. In general, in terms of gender differences, the significant changes indicated by the composition and abundance of the gut microbiota in females are more complex and diverse than those in males, and the significantly changed pathways enriched in females and males seem to be almost completely different. In terms of individual differences, the significantly changed pathways indicated by the gut microbiota data of larger individuals are more diverse, but certain similar changes are still maintained; for example, the endocrine system was significantly up-regulated in both larger female and male individuals, compared with smaller females and males (Supplementary Table S3). On the other hand, the intestinal microbiota of larger-sized male crayfishes seem to be particularly involved in the pathways related to immune system diseases, suggesting that the larger male crayfishes are possibly more susceptible to pathogenic invasion. The composition and abundance of gut microbiota in larger female individuals may have selectively enhanced energy metabolism (Supplementary Table S3).

In the present study, key differences in the gut flora of crayfishes with various sizes and genders were investigated. Given the profound influence of environment on microbiota, environmental variability may dilute or even mask shifts in gut microbiota driven by differences in crayfish body size or sex. Thus, the consistent farming conditions were strictly maintained in this study to mitigate such effects. However, several additional factors should be carefully considered in experimental design, result analysis, and discussion. The locomotor ability, activity range, and competitive advantages (or disadvantages) in feeding among individuals of varying sizes, along with variations in hormone types, secretion levels, metabolism, and immunity across developmental stages, should be taken into account when exploring how individual size influences gut microbiota. Furthermore, sexual dimorphism, variations in physiological developmental stages, and associated differences in metabolic and immune traits should be incorporated into analysis when the effects of animal sex on gut microbiota are investigated. Additionally, given the strong influence of environment on microbiota, the combined effects of environmental factors and individual traits of farmed animals (e.g., body size and sex) on the composition of gut microbiota should be addressed in subsequent studies.

## 5. Conclusions 

In this study, the gut microbiota of red-claw crayfish (*C. quadricarinatus*) of different body sizes and genders was characterized using 16S rRNA gene sequencing. The results revealed similarities and differences in the intestinal microbial communities among individuals of varying sizes and genders reared under consistent farming conditions, indicating that body size and gender are potentially important factors that independently, or jointly, shape the composition and abundance of gut microbiota in this species. This study is expected to contribute to improving aquaculture yield and production efficiency in red-claw crayfish farming, while providing a solid foundation for the development and application of probiotics.

## Figures and Tables

**Table 1:** The 16S rRNA gene sequencing statistics and basic taxonomic information of all the samples.

Group.	Sample	Trimmed Tags	ASV Number	Phylum	Class	Order	Family	Genus
GUBF	GUBF2	79024	272	17	30	66	109	170
	GUBF3	78901	357	21	37	78	135	210
	GUBF4	76053	628	23	57	121	200	336
	GUBF5	76014	558	22	52	112	179	304
	GUBF6	75887	483	22	35	83	142	266
	Mean	77176	460	21	42	92	153	257
GUBM	GUBM1	75170	577	23	42	101	169	303
	GUBM2	74851	563	22	38	90	162	296
	GUBM3	74418	509	21	39	79	146	263
	GUBM4	74110	130	13	19	42	69	95
	GUBM6	73985	350	18	28	67	114	206
	Mean	74507	426	19	33	76	132	233
GUSF	GUSF1	73943	206	17	26	59	98	134
	GUSF2	73676	264	22	31	68	114	164
	GUSF3	73587	320	22	36	69	109	173
	GUSF5	73030	211	14	24	52	74	114
	GUSF6	72986	250	14	21	43	68	96
	Mean	73444	250	18	28	58	93	136
GUSM	GUSM1	72252	193	13	20	46	73	111
	GUSM2	71996	210	14	22	40	62	89
	GUSM3	70958	280	19	34	71	121	178
	GUSM4	70719	206	15	25	49	82	126
	GUSM6	70666	273	12	20	40	68	98
	Mean	71318	232	15	24	49	81	120

**Table 2:** The proportion of the dominant phyla and genera (Proportion > 1%) in the intestinal microbiota of different crayfish groups.

GUSM		GUBM		GUSF		GUBF	
**Phylum**	Proportion (%)	Phylum	Proportion (%)	Phylum	Proportion (%)	Phylum	Proportion (%)
*Firmicutes*	55.10	*Firmicutes*	70.62	*Firmicutes*	52.79	*Proteobacteria*	45.73
*Proteobacteria*	39.15	*Proteobacteria*	20.62	*Proteobacteria*	38.88	*Firmicutes*	22.10
*Unclassified*	1.65	*Bacteroidota*	2.24	*Fusobacteriota*	2.08	*Bacteroidota*	10.52
*Fusobacteriota*	1.58	*Fusobacteriota*	2.08	*Deinococcota*	1.61	*Deinococcota*	4.66
		*Deinococcota*	1.65	*Planctomycetota*	1.25	*Fusobacteriota*	3.49
				*Bacteroidota*	1.23	*Unclassified*	3.16
						*Actinobacteriota*	2.34
						*Cyanobacteria*	2.09
						*Planctomycetota*	1.80
Total	97.49		97.21		97.85		95.88
**Genus**	**Proportion** **(%)**	**Genus**	**Proportion** **(%)**	**Genus**	**Proportion** **(%)**	**Genus**	**Proportion** **(%)**
*Citrobacter*	30.43	*Candidatus_Hepatoplasma*	42.28	*Tyzzerella*	24.29	*Vibrio*	10.50
*Candidatus_Bacilloplasma*	28.03	*Vibrio*	13.55	*Candidatus_Bacilloplasma*	16.70	*Tyzzerella*	7.74
*Tyzzerella*	15.65	*Candidatus_Bacilloplasma*	11.98	*Vibrio*	15.10	*Candidatus_Bacilloplasma*	7.49
*Candidatus_Hepatoplasma*	5.73	*Lactovum*	5.77	*Citrobacter*	7.77	*Deinococcus*	4.19
*Mycoplasmataceae_unclassified*	3.40	*Tyzzerella*	4.75	*Candidatus_Hepatoplasma*	5.62	*Betaproteobacteria_unclassified*	3.63
*Vibrio*	3.09	*Mycoplasmataceae_unclassified*	3.02	*Mycoplasmataceae_unclassified*	3.26	*Hypnocyclicus*	3.40
*Acinetobacter*	2.70	*Hypnocyclicus*	1.32	*Rhodobacteraceae_unclassified*	2.36	*Unclassified*	3.16
*Unclassified*	1.65	*Deinococcus*	1.24	*Bosea*	2.36	*AAP99*	2.64
*Hypnocyclicus*	1.42			*Hypnocyclicus*	2.02	*Arcicella*	2.48
				*Candidatus_Hepatincola_unclassified*	1.94	*Mycoplasmataceae_unclassified*	2.41
				*Deinococcus*	1.59	*Rhodobacteraceae_unclassified*	2.39
				*Pseudomonas*	1.22	*Chloroplast_unclassified*	2.07
				*Fimbriiglobus*	1.04	*Citrobacter*	1.75
						*Env.OPS_17_unclassified*	1.55
						*Pelomonas*	1.46
						*Lactovum*	1.46
						*Acinetobacter*	1.29
						*Rhodobacter*	1.25
						*Candidatus_Hepatincola_unclassified*	1.19
						*Fimbriiglobus*	1.18
						*Pseudomonas*	1.14
						*Candidatus_Hepatoplasma*	1.10
Total	92.10		83.92		85.27		65.48

**Table 3:** The significantly abundant phyla and genera in the intestinal microbiota of different crayfish groups.

**GUBF** **vs.** **GUBM**	**Phylum**	**Mean** **_GUBF**	**Std** **_GUBF**	**Mean** **_GUBM**	**Std** **_GUBM**	***p*** **value**	**Regulation**
	*Acidobacteriota*	0.84819	0.68453	0.01432	0.02220	0.00900	up
	*Myxococcota*	0.71460	0.39075	0.05061	0.06951	0.01630	up
	*Campylobacterota*	0.00000	0.00000	0.04553	0.07459	0.01860	down
	*Firmicutes*	22.09640	26.12140	70.61692	17.48706	0.02830	down
	*Dependentiae*	0.20797	0.23477	0.00217	0.00486	0.03430	up
	*Bacteroidota*	10.51743	9.02314	2.24308	3.11525	0.04720	up
	*Planctomycetota*	1.80193	1.36699	0.26451	0.24415	0.04720	up
**GUBF** **vs.** **GUSF**	**Phylum**	**Mean** **_GUBF**	**Std** **_GUBF**	**Mean** **_GUSF**	**Std** **_GUSF**	***p*** **value**	**Regulation**
	*Campylobacterota*	0.00000	0.00000	0.01363	0.01335	0.00530	down
	*Acidobacteriota*	0.84819	0.68453	0.01954	0.03793	0.01500	up
	*Myxococcota*	0.71460	0.39075	0.03271	0.05154	0.01600	up
	*Bacteroidota*	10.51743	9.02314	1.22784	1.66926	0.02830	up
	*Cyanobacteria*	2.09333	2.02594	0.06871	0.10044	0.02830	up
	*Firmicutes*	22.09640	26.12140	52.79242	22.42735	0.04720	down
**GUBM** **vs.** **GUSM**	**Phylum**	**Mean** **_GUBM**	**Std** **_GUBM**	**Mean** **_GUSM**	**Std** **_GUSM**	***p*** **value**	**Regulation**
	*Chloroflexi*	0.02217	0.01431	0.00082	0.00184	0.03430	up
	*Verrucomicrobiota*	0.49829	0.28979	0.10280	0.06334	0.04720	up
**GUSF** **vs.** **GUSM**	**Phylum**	**Mean** **_GUSF**	**Std** **_GUSF**	**Mean** **_GUSM**	**Std** **_GUSM**	***p*** **value**	**Regulation**
	*Dependentiae*	0.03433	0.06569	0.00000	0.00000	0.01860	up
	*Actinobacteriota*	0.72913	1.05930	0.09432	0.11858	0.02830	up
	*Chloroflexi*	0.03541	0.04792	0.00082	0.00184	0.03430	up
	*Planctomycetota*	1.25159	2.40843	0.06625	0.11366	0.04720	up
**GUBF** **vs.** **GUBM**	**Genus**	**Mean_GUBF**	**Std_GUBF**	**Mean_GUBM**	**Std_GUBM**	***p*** **value**	**Regulation**
	*Pelomonas*	1.46167	1.44935	0.00000	0.00000	0.00530	up
	*Anaerorhabdus_furcosa_group*	0.00000	0.00000	0.09288	0.11232	0.00530	down
	*Sphingobacteriales_unclassified*	0.16251	0.18890	0.00000	0.00000	0.00530	up
	*Acidovorax*	0.37621	0.28762	0.00054	0.00122	0.00710	up
	*UKL13-1*	0.08288	0.09078	0.00053	0.00119	0.00710	up
	*NS11-12_marine_group_unclassified*	0.03436	0.02169	0.00190	0.00425	0.00710	up
	*Bryobacter*	0.40512	0.21599	0.00137	0.00193	0.00820	up
	*Arcicella*	2.47547	2.24407	0.02059	0.03411	0.00900	up
	*Bacteroides*	0.00929	0.00824	0.15106	0.17442	0.00900	down
	*Vogesella*	0.50191	0.62873	0.00210	0.00470	0.01320	up
	*Dysgonomonas*	0.00164	0.00367	0.13542	0.23667	0.01320	down
	*mle1-27_unclassified*	0.10478	0.07991	0.00244	0.00412	0.01500	up
	*Kinneretia*	0.84167	0.74135	0.00693	0.00684	0.01600	up
	*Saprospiraceae_unclassified*	0.36534	0.37424	0.01242	0.01869	0.01600	up
	*Rhodobacteraceae_unclassified*	2.38594	2.05941	0.18528	0.24128	0.01630	up
	*Novosphingobium*	0.91230	0.48971	0.18149	0.33630	0.01630	up
	*Pirellula*	0.34579	0.39889	0.00000	0.00000	0.01860	up
	*Acetobacter*	0.08312	0.15248	0.00000	0.00000	0.01860	up
	*Solirubrobacterales_unclassified*	0.07894	0.16792	0.00000	0.00000	0.01860	up
	*Lactococcus*	0.00000	0.00000	0.06547	0.12113	0.01860	down
	*Kaistia*	0.00000	0.00000	0.05207	0.09577	0.01860	down
	*Sphingomonadaceae_unclassified*	0.03356	0.03207	0.00000	0.00000	0.01860	up
	*Sphingopyxis*	0.03237	0.02919	0.00000	0.00000	0.01860	up
	*PLTA13_unclassified*	0.03018	0.04026	0.00000	0.00000	0.01860	up
	*WCHB1-32*	0.00000	0.00000	0.01850	0.01645	0.01860	down
	*Helicobacter*	0.00000	0.00000	0.00381	0.00359	0.01860	down
	*Coprococcus*	0.00000	0.00000	0.01053	0.00881	0.01860	down
	*Plesiomonas*	0.00000	0.00000	0.00699	0.00541	0.01860	down
	*Rhodocytophaga*	0.00000	0.00000	0.00514	0.00377	0.01860	down
	*Clostridium_sensu_stricto_1*	0.03105	0.06942	0.13700	0.07735	0.02360	down
	*Edaphobaculum*	0.36533	0.23754	0.00813	0.01606	0.02640	up
	*Lacihabitans*	0.24392	0.19627	0.01286	0.01863	0.02640	up
	*AAP99*	2.64359	2.82013	0.20975	0.37848	0.02830	up
	*Flavobacterium*	0.71153	0.58629	0.03785	0.04972	0.02830	up
	*env.OPS_17_unclassified*	1.55415	1.51370	0.06336	0.12440	0.02830	up
	*Burkholderiales_unclassified*	0.88513	0.50714	0.08069	0.10787	0.02830	up
	*Sediminibacterium*	0.78644	0.66903	0.04466	0.08275	0.02830	up
	*Phreatobacter*	0.58423	0.45650	0.02609	0.03664	0.02830	up
	*Akkermansia*	0.08922	0.11654	0.30065	0.14112	0.02830	down
	*Haliangium*	0.17125	0.14827	0.02789	0.04857	0.02830	up
	*Rheinheimera*	0.65472	0.59817	0.00134	0.00299	0.03430	up
	*Babeliaceae_unclassified*	0.19694	0.22618	0.00217	0.00486	0.03430	up
	*Neisseriaceae_unclassified*	0.15191	0.22398	0.00053	0.00119	0.03430	up
	*Mitochondria_unclassified*	0.04877	0.04003	0.00079	0.00176	0.03430	up
	*Enhydrobacter*	0.02515	0.04311	0.00080	0.00179	0.03430	up
	*Prevotella_9*	0.00158	0.00353	0.01028	0.00616	0.03430	down
	*Christensenellaceae_R-7_group*	0.00132	0.00294	0.01079	0.00626	0.03430	down
	*Subdoligranulum*	0.00132	0.00294	0.01022	0.00600	0.03430	down
	*Pajaroellobacter*	0.21550	0.20625	0.01617	0.01732	0.04650	up
	*Ensifer*	0.11633	0.08993	0.01271	0.01162	0.04650	up
	*Candidatus_Hepatoplasma*	1.10041	1.08793	42.28378	33.81319	0.04720	down
	*Betaproteobacteria_unclassified*	3.63215	2.93863	0.58376	0.81851	0.04720	up
	*Rhizobium*	0.79042	0.65016	0.18267	0.29645	0.04720	up
	*Sphingomonas*	0.43796	0.31654	0.03780	0.02199	0.04720	up
	*Piscinibacter*	0.41842	0.35342	0.05036	0.08130	0.04720	up
**GUBF** **vs.** **GUSF**	**Genus**	**Mean** **_GUBF**	**Std** **_GUBF**	**Mean** **_GUSF**	**Std** **_GUSF**	***p*** **value**	**Regulation**
	*Acidovorax*	0.37621	0.28762	0.00000	0.00000	0.00530	up
	*Sphingobacteriales_unclassified*	0.16251	0.18890	0.00000	0.00000	0.00530	up
	*Dechloromonas*	0.03440	0.03016	0.00000	0.00000	0.00530	up
	*NS11-12_marine_group_unclassified*	0.03436	0.02169	0.00000	0.00000	0.00530	up
	*Helicobacter*	0.00000	0.00000	0.00804	0.00211	0.00530	down
	*Arcicella*	2.47547	2.24407	0.00905	0.02023	0.00710	up
	*Pelomonas*	1.46167	1.44935	0.00054	0.00121	0.00710	up
	*Kinneretia*	0.84167	0.74135	0.00163	0.00364	0.00710	up
	*Bryobacter*	0.40512	0.21599	0.00081	0.00182	0.00710	up
	*Lacihabitans*	0.24392	0.19627	0.00080	0.00178	0.00710	up
	*Haliangium*	0.17125	0.14827	0.00612	0.01368	0.00710	up
	*Saprospiraceae_unclassified*	0.36534	0.37424	0.00403	0.00605	0.00820	up
	*Sediminibacterium*	0.78644	0.66903	0.00561	0.00960	0.00880	up
	*37-13_unclassified*	0.29714	0.31751	0.00296	0.00336	0.00880	up
	*Betaproteobacteria_unclassified*	3.63215	2.93863	0.18412	0.20016	0.00900	up
	*Novosphingobium*	0.91230	0.48971	0.03744	0.03807	0.00900	up
	*Burkholderiales_unclassified*	0.88513	0.50714	0.02018	0.01718	0.00900	up
	*Bacteroidota_unclassified*	0.23968	0.23327	0.00378	0.00846	0.01320	up
	*Alkanindiges*	0.07742	0.06256	0.00293	0.00654	0.01320	up
	*env.OPS_17_unclassified*	1.55415	1.51370	0.02868	0.05179	0.01500	up
	*Lachnospiraceae_unclassified*	0.03797	0.03483	0.00454	0.00534	0.01600	up
	*Citrobacter*	1.75451	2.58324	7.76702	4.72260	0.01630	down
	*AAP99*	2.64359	2.82013	0.09300	0.11008	0.01630	up
	*Rheinheimera*	0.65472	0.59817	0.00000	0.00000	0.01860	up
	*Stenotrophomonas*	0.04828	0.06762	0.00000	0.00000	0.01860	up
	*Spirosomaceae_unclassified*	0.26214	0.28389	0.00000	0.00000	0.01860	up
	*Neisseriaceae_unclassified*	0.15191	0.22398	0.00000	0.00000	0.01860	up
	*Streptococcus*	0.01401	0.01859	0.00000	0.00000	0.01860	up
	*Rhodoferax*	0.06657	0.05177	0.00000	0.00000	0.01860	up
	*Acetobacter*	0.08312	0.15248	0.00000	0.00000	0.01860	up
	*Solirubrobacterales_unclassified*	0.07894	0.16792	0.00000	0.00000	0.01860	up
	*Mitochondria_unclassified*	0.04877	0.04003	0.00000	0.00000	0.01860	up
	*Sphingomonadaceae_unclassified*	0.03356	0.03207	0.00000	0.00000	0.01860	up
	*Sphingopyxis*	0.03237	0.02919	0.00000	0.00000	0.01860	up
	*PLTA13_unclassified*	0.03018	0.04026	0.00000	0.00000	0.01860	up
	*Ramlibacter*	0.02023	0.02394	0.00000	0.00000	0.01860	up
	*Butyricicoccus*	0.00552	0.00327	0.00000	0.00000	0.01860	up
	*Rikenellaceae_RC9_gut_group*	0.00412	0.00237	0.00000	0.00000	0.01860	up
	*mle1-27_unclassified*	0.10478	0.07991	0.01384	0.03096	0.02360	up
	*Edaphobaculum*	0.36533	0.23754	0.00188	0.00262	0.02640	up
	*Pajaroellobacter*	0.21550	0.20625	0.01194	0.02117	0.02640	up
	*Comamonadaceae_unclassified*	0.10474	0.09014	0.00650	0.01013	0.02640	up
	*Bifidobacterium*	0.03257	0.02362	0.00371	0.00578	0.02640	up
	*Rhizobium*	0.79042	0.65016	0.12497	0.24357	0.02780	up
	*Deinococcaceae_unclassified*	0.44655	0.37977	0.02540	0.03923	0.02780	up
	*Chloroplast_unclassified*	2.07102	2.03471	0.06818	0.09932	0.02830	up
	*Emticicia*	0.84730	0.86335	0.00516	0.01153	0.03430	up
	*Pantoea*	0.17450	0.18215	0.01977	0.04420	0.04060	up
	*Rurimicrobium*	0.01065	0.02167	0.08329	0.13866	0.04450	down
	*Vogesella*	0.50191	0.62873	0.01949	0.03429	0.04650	up
	*Piscinibacter*	0.41842	0.35342	0.04197	0.07214	0.04650	up
	*UCG-005*	0.02842	0.01702	0.00616	0.00571	0.04650	up
	*Brevundimonas*	0.50008	0.26627	0.09912	0.15847	0.04720	up
	*Hydrogenophaga*	0.33209	0.21457	0.08131	0.11985	0.04720	up
	*Ferruginibacter*	0.22315	0.21484	0.04605	0.08226	0.04720	up
**GUBM** **vs.** **GUSM**	**Genus**	**Mean** **_GUBM**	**Std** **_GUBM**	**Mean** **_GUSM**	**Std** **_GUSM**	***p*** **value**	**Regulation**
	*Cavicella*	0.02117	0.01518	0.00056	0.00124	0.00710	up
	*Romboutsia*	0.06026	0.03129	0.00575	0.00789	0.00820	up
	*Akkermansia*	0.30065	0.14112	0.03252	0.01007	0.00900	up
	*Shewanella*	0.03391	0.01437	0.00780	0.00511	0.00900	up
	*Novosphingobium*	0.18149	0.33630	0.00569	0.00908	0.01500	up
	*Bacteroides*	0.15106	0.17442	0.01516	0.01865	0.01630	up
	*Piscinibacter*	0.05036	0.08130	0.00000	0.00000	0.01860	up
	*Prosthecobacter*	0.04714	0.06719	0.00000	0.00000	0.01860	up
	*Curtobacterium*	0.11285	0.06482	0.00000	0.00000	0.01860	up
	*Faecalibacterium*	0.07804	0.04573	0.00000	0.00000	0.01860	up
	*Rodentibacter*	0.07227	0.04900	0.00000	0.00000	0.01860	up
	*Clostridia_UCG-014_unclassified*	0.05925	0.04104	0.00000	0.00000	0.01860	up
	*Noviherbaspirillum*	0.05311	0.03288	0.00000	0.00000	0.01860	up
	*Azospirillum*	0.03612	0.02212	0.00000	0.00000	0.01860	up
	*Microvirga*	0.03062	0.02047	0.00000	0.00000	0.01860	up
	*Erwinia*	0.02647	0.01645	0.00000	0.00000	0.01860	up
	*Orrella*	0.02182	0.01462	0.00000	0.00000	0.01860	up
	*Streptomyces*	0.02099	0.01200	0.00000	0.00000	0.01860	up
	*Terrisporobacter*	0.01080	0.00670	0.00000	0.00000	0.01860	up
	*WCHB1-32*	0.01850	0.01645	0.00000	0.00000	0.01860	up
	*AKIW781_unclassified*	0.01348	0.00983	0.00000	0.00000	0.01860	up
	*Ferrovibrionales_unclassified*	0.01186	0.00727	0.00000	0.00000	0.01860	up
	*Christensenellaceae_R-7_group*	0.01079	0.00626	0.00000	0.00000	0.01860	up
	*Tuzzerella*	0.00836	0.00518	0.00000	0.00000	0.01860	up
	*Enterorhabdus*	0.00811	0.00552	0.00000	0.00000	0.01860	up
	*Veillonella*	0.00568	0.00479	0.00000	0.00000	0.01860	up
	*Prevotellaceae_NK3B31_group*	0.00697	0.00542	0.00000	0.00000	0.01860	up
	*Plesiomonas*	0.00699	0.00541	0.00000	0.00000	0.01860	up
	*Rhodocytophaga*	0.00514	0.00377	0.00000	0.00000	0.01860	up
	*Chryseobacterium*	0.11417	0.14953	0.00328	0.00603	0.02640	up
	*Enterococcus*	0.05551	0.03335	0.00283	0.00363	0.02780	up
	*Eubacterium coprostanoligenes_group_unclassified*	0.03973	0.02847	0.00437	0.00502	0.02780	up
	*Citrobacter*	0.57602	0.74011	30.42947	25.79339	0.02830	down
	*Caproiciproducens*	0.07592	0.05369	0.01318	0.01230	0.02830	up
	*Clostridium_sensu_stricto_1*	0.13700	0.07735	0.01043	0.01361	0.02830	up
	*Devosia*	0.01024	0.01109	0.00051	0.00113	0.03430	up
	*Dorea*	0.02629	0.01808	0.00137	0.00306	0.03430	up
	*Ruminococcaceae_unclassified*	0.02686	0.01781	0.00051	0.00113	0.03430	up
	*Rothia*	0.02481	0.01604	0.00055	0.00123	0.03430	up
	*Intestinimonas*	0.01484	0.00898	0.00055	0.00123	0.03430	up
	*Paramuribaculum*	0.00937	0.00695	0.00101	0.00226	0.03430	up
	*Negativibacillus*	0.00784	0.00505	0.00054	0.00121	0.03430	up
	*Coprococcus*	0.01053	0.00881	0.00082	0.00184	0.03430	up
	*Subdoligranulum*	0.01022	0.00600	0.00051	0.00113	0.03430	up
	*Oscillospiraceae_unclassified*	0.00707	0.00653	0.00051	0.00113	0.03430	up
	*Bilophila*	0.00590	0.00403	0.00056	0.00124	0.03430	up
	*Candidatus_Hepatoplasma*	42.28378	33.81319	5.72612	7.40385	0.04720	up
	*Escherichia-Shigella*	0.22632	0.12115	0.06271	0.02727	0.04720	up
**GUSF** **vs.** **GUSM**	**Genus**	**Mean** **_GUSF**	**Std** **_GUSF**	**Mean** **_GUSM**	**Std** **_GUSM**	***p*** **value**	**Regulation**
	*Babeliaceae_unclassified*	0.03433	0.06569	0.00000	0.00000	0.01860	up
	*Legionella*	0.05593	0.10938	0.00055	0.00123	0.03430	up
	*Fimbriiglobus*	1.03971	1.98544	0.04362	0.07290	0.04720	up
	*Actinobacteriota_unclassified*	0.22440	0.38076	0.03110	0.06306	0.04720	up

## Data Availability

Dataset available on request from the authors.

## Supplementary Materials

The following supporting information can be downloaded at: https://www.mdpi.com/article/10.3390/biology14091209/s1, Table S1: The average body weight, length and width of the individual crayfishes in the larger-sized female (BF), larger-sized male (BM), smaller-sized female (SF), and small-er-sized male (SM) groups; Table S2: The significantly different phyla and genera in abundance among all the experimental groups; Table S3: The significantly regulated pathways predicted by PICRUSt2 based on KEGG database in the pairwise comparisons of GUBF vs. GUBM, GUSF vs. GUSM, GUBF vs. GUSF, and GUBM vs. GUSM; Figure S1: The Manhattan plot displaying the significantly enriched or depleted intestinal microbial phylum in abundance with a pairwise comparison pattern.
