# Supplementary material for: Key Differences in the Gut Microbiota of Red-Claw Crayfish Cherax quadricarinatus with Different Sizes and Genders Under Consistent Farming Conditions
Source: Biology (Basel). 2025 Sep 7;14(9):1209. doi: 10.3390/biology14091209 (PMC12467359; doi:10.3390/biology14091209)
Supplement: Supplementary file 1 [file biology-14-01209-s001.zip › biology-3824440-supplementary.pdf]

Table S1 The average body weight, length and width of the individual crayfishes in the larger-sized female (BF), larger-sized male (BM), smaller-sized female (SF), and small-er-sized male (SM) groups.

Group	Larger-sized female (BF)		Larger-sized male (BM)		Smaller-sized female (SF)		Smaller-sized male (SM)		BF vs BM	BF vs SF	BM vs SM	SF vs SM
	Mean	Std	Mean	Std	Mean	Std	Mean	Std	*P*-value	*P*-value	*P*-value	*P*-value
Body Lenth (cm)	13.43636	0.45227	14.00000	1.06941	11.35556	0.65549	10.70000	0.72761	0.11552	1.42E-10	2.85E-08	0.00760
Body Width (cm)	2.86364	0.18040	3.15000	0.30302	2.35556	0.17564	2.32778	0.16380	0.01228	2.76E-07	2.97E-07	0.62680
Weight (g)	51.35455	5.66399	67.23333	14.46144	25.85556	4.98565	26.37222	4.68989	0.00322	1.51E-10	4.51E-07	0.75075

Table S2 The significantly different phyla and genera in abundance among all the experimental groups.

	**GUBF**		**GUBM**		**GUSF**		**GUSM**		***P* value**
	Mean	Std	Mean	Std	Mean	Std	Mean	Std	
Phylum									
Myxococcota	0.71460	0.39075	0.05061	0.06951	0.03271	0.05154	0.00856	0.01776	0.01080
Actinobacteriota	2.33966	1.96630	0.56460	0.47637	0.72913	1.05930	0.09432	0.11858	0.01370
Chloroflexi	0.10456	0.08162	0.02217	0.01431	0.03541	0.04792	0.00082	0.00184	0.01600
Acidobacteriota	0.84819	0.68453	0.01432	0.02220	0.01954	0.03793	0.01266	0.02491	0.01630
Dependentiae	0.20797	0.23477	0.00217	0.00486	0.03433	0.06569	0.00000	0.00000	0.01790

Bacteroidota	10.51743	9.02314	2.24308	3.11525	1.22784	1.66926	0.94541	1.82471	0.02670
Campylobacterota	0.00000	0.00000	0.04553	0.07459	0.01363	0.01335	0.00503	0.00497	0.03280
Verrucomicrobiota	0.63546	0.21835	0.49829	0.28979	0.30527	0.41891	0.10280	0.06334	0.03950
Planctomycetota	1.80193	1.36699	0.26451	0.24415	1.25159	2.40843	0.06625	0.11366	0.04880
Genus									
Acidovorax	0.37621	0.28762	0.00054	0.00122	0.00000	0.00000	0.00000	0.00000	0.00080
Sphingobacteriales_unclassified	0.16251	0.18890	0.00000	0.00000	0.00000	0.00000	0.00083	0.00186	0.00120
Pelomonas	1.46167	1.44935	0.00000	0.00000	0.00054	0.00121	0.00758	0.00973	0.00180
NS11-12_marine_group_unclassified	0.03436	0.02169	0.00190	0.00425	0.00000	0.00000	0.00217	0.00361	0.00240
Novosphingobium	0.91230	0.48971	0.18149	0.33630	0.03744	0.03807	0.00569	0.00908	0.00290
Acetobacter	0.08312	0.15248	0.00000	0.00000	0.00000	0.00000	0.00000	0.00000	0.00290
Solirubrobacterales_unclassified	0.07894	0.16792	0.00000	0.00000	0.00000	0.00000	0.00000	0.00000	0.00290
Sphingopyxis	0.03237	0.02919	0.00000	0.00000	0.00000	0.00000	0.00000	0.00000	0.00290
PLTA13_unclassified	0.03018	0.04026	0.00000	0.00000	0.00000	0.00000	0.00000	0.00000	0.00290
WCHB1-32	0.00000	0.00000	0.01850	0.01645	0.00000	0.00000	0.00000	0.00000	0.00290
Plesiomonas	0.00000	0.00000	0.00699	0.00541	0.00000	0.00000	0.00000	0.00000	0.00290
Rhodocytophaga	0.00000	0.00000	0.00514	0.00377	0.00000	0.00000	0.00000	0.00000	0.00290
Bryobacter	0.40512	0.21599	0.00137	0.00193	0.00081	0.00182	0.00500	0.01118	0.00460
Arcicella	2.47547	2.24407	0.02059	0.03411	0.00905	0.02023	0.05286	0.11257	0.00610
Saprospiraceae_unclassified	0.36534	0.37424	0.01242	0.01869	0.00403	0.00605	0.00083	0.00186	0.00610
mle1-27_unclassified	0.10478	0.07991	0.00244	0.00412	0.01384	0.03096	0.00000	0.00000	0.00610
Sediminibacterium	0.78644	0.66903	0.04466	0.08275	0.00561	0.00960	0.00055	0.00123	0.00620
Kinneretia	0.84167	0.74135	0.00693	0.00684	0.00163	0.00364	0.01000	0.00705	0.00720
Citrobacter	1.75451	2.58324	0.57602	0.74011	7.76702	4.72260	30.42947	25.79339	0.00760
Rheinheimera	0.65472	0.59817	0.00134	0.00299	0.00000	0.00000	0.00000	0.00000	0.00780
Neisseriaceae_unclassified	0.15191	0.22398	0.00053	0.00119	0.00000	0.00000	0.00000	0.00000	0.00780

Christensenellaceae_R-7_group	0.00132	0.00294	0.01079	0.00626	0.00000	0.00000	0.00000	0.00000	0.00780
Haliangium	0.17125	0.14827	0.02789	0.04857	0.00612	0.01368	0.00806	0.01801	0.00800
Piscinibacter	0.41842	0.35342	0.05036	0.08130	0.04197	0.07214	0.00000	0.00000	0.00870
Betaproteobacteria_unclassified	3.63215	2.93863	0.58376	0.81851	0.18412	0.20016	0.05301	0.08929	0.00900
Dechloromonas	0.03440	0.03016	0.02950	0.05872	0.00000	0.00000	0.00152	0.00340	0.00900
Lacihabitans	0.24392	0.19627	0.01286	0.01863	0.00080	0.00178	0.00306	0.00683	0.00920
Akkermansia	0.08922	0.11654	0.30065	0.14112	0.03239	0.00838	0.03252	0.01007	0.00990
Vogesella	0.50191	0.62873	0.00210	0.00470	0.01949	0.03429	0.00076	0.00170	0.01000
Azospirillum	0.00763	0.01706	0.03612	0.02212	0.00000	0.00000	0.00000	0.00000	0.01060
Ferrovibrionales_unclassified	0.00237	0.00530	0.01186	0.00727	0.00000	0.00000	0.00000	0.00000	0.01060
Enterorhabdus	0.00132	0.00294	0.00811	0.00552	0.00000	0.00000	0.00000	0.00000	0.01060
Anaerorhabdus_furcosa_group	0.00000	0.00000	0.09288	0.11232	0.84063	1.87823	0.13230	0.21515	0.01110
Comamonadaceae_unclassified	0.10474	0.09014	0.04552	0.08020	0.00650	0.01013	0.00000	0.00000	0.01210
Bacteroidota_unclassified	0.23968	0.23327	0.03926	0.05046	0.00378	0.00846	0.00680	0.00781	0.01350
Cavicella	0.34077	0.43687	0.02117	0.01518	0.00160	0.00357	0.00056	0.00124	0.01400
Sphingomonadaceae_unclassified	0.03356	0.03207	0.00000	0.00000	0.00000	0.00000	0.01167	0.02609	0.01400
Microvirga	0.00737	0.01647	0.03062	0.02047	0.00000	0.00000	0.00000	0.00000	0.01400
Tuzzerella	0.00237	0.00530	0.00836	0.00518	0.00000	0.00000	0.00000	0.00000	0.01400
Bacteroides	0.00929	0.00824	0.15106	0.17442	0.00612	0.00556	0.01516	0.01865	0.01440
Prosthecobacter	0.22947	0.18428	0.04714	0.06719	0.06283	0.08690	0.00000	0.00000	0.01490
Ensifer	0.11633	0.08993	0.01271	0.01162	0.03217	0.07193	0.00000	0.00000	0.01500
Rhodoferax	0.06657	0.05177	0.01621	0.01320	0.00000	0.00000	0.00166	0.00248	0.01530
Pajaroellobacter	0.21550	0.20625	0.01617	0.01732	0.01194	0.02117	0.00051	0.00113	0.01560
Clostridia_UCG-014_unclassified	0.01792	0.03782	0.05925	0.04104	0.00000	0.00000	0.00000	0.00000	0.01600
Subdoligranulum	0.00132	0.00294	0.01022	0.00600	0.00000	0.00000	0.00051	0.00113	0.01760
Babeliaceae_unclassified	0.19694	0.22618	0.00217	0.00486	0.03433	0.06569	0.00000	0.00000	0.01790

Curtobacterium	0.03078	0.06883	0.11285	0.06482	0.00000	0.00000	0.00000	0.00000	0.01820
Noviherbaspirillum	0.01342	0.03000	0.05311	0.03288	0.00000	0.00000	0.00000	0.00000	0.01820
Erwinia	0.00710	0.01589	0.02647	0.01645	0.00000	0.00000	0.00000	0.00000	0.01820
Orrella	0.00631	0.01412	0.02182	0.01462	0.00000	0.00000	0.00000	0.00000	0.01820
AKIW781_unclassified	0.00500	0.01118	0.01348	0.00983	0.00000	0.00000	0.00000	0.00000	0.01820
Prevotellaceae_NK3B31_group	0.00184	0.00412	0.00697	0.00542	0.00000	0.00000	0.00000	0.00000	0.01820
P3OB-42	0.22119	0.35613	0.00000	0.00000	0.00000	0.00000	0.00000	0.00000	0.01870
Flectobacillus	0.10277	0.12570	0.00000	0.00000	0.00000	0.00000	0.00000	0.00000	0.01870
Gaiellales_unclassified	0.03914	0.03582	0.00000	0.00000	0.00000	0.00000	0.00000	0.00000	0.01870
Candidatus_Midichloria	0.03361	0.03842	0.00000	0.00000	0.00000	0.00000	0.00000	0.00000	0.01870
Leifsonia	0.01582	0.02168	0.00000	0.00000	0.00000	0.00000	0.00000	0.00000	0.01870
RsaHf231_unclassified	0.00000	0.00000	0.01472	0.01783	0.00000	0.00000	0.00000	0.00000	0.01870
Actinomyces	0.01187	0.01116	0.00000	0.00000	0.00000	0.00000	0.00000	0.00000	0.01870
Vermiphilaceae_unclassified	0.01103	0.01387	0.00000	0.00000	0.00000	0.00000	0.00000	0.00000	0.01870
Candidatus_Ovatusbacter	0.00960	0.00928	0.00000	0.00000	0.00000	0.00000	0.00000	0.00000	0.01870
Sporichthyaceae_unclassified	0.00000	0.00000	0.00427	0.00546	0.00000	0.00000	0.00000	0.00000	0.01870
Skermanella	0.00000	0.00000	0.00409	0.00412	0.00000	0.00000	0.00000	0.00000	0.01870
Steroidobacter	0.00000	0.00000	0.00324	0.00349	0.00000	0.00000	0.00000	0.00000	0.01870
Niabella	0.00280	0.00263	0.00000	0.00000	0.00000	0.00000	0.00000	0.00000	0.01870
Roseisolibacter	0.00000	0.00000	0.00272	0.00289	0.00000	0.00000	0.00000	0.00000	0.01870
Burkholderiales_unclassified	0.88513	0.50714	0.08069	0.10787	0.02018	0.01718	0.03068	0.04401	0.01880
Dysgonomonas	0.00164	0.00367	0.13542	0.23667	0.02542	0.04413	0.03500	0.02735	0.01930
Faecalibacterium	0.02242	0.04781	0.07804	0.04573	0.00000	0.00000	0.00000	0.00000	0.01930
UKL13-1	0.08288	0.09078	0.00053	0.00119	0.00973	0.01646	0.00611	0.01367	0.02060
Pirellula	0.34579	0.39889	0.00000	0.00000	0.00893	0.01522	0.00127	0.00283	0.02160
Romboutsia	0.02443	0.02977	0.06026	0.03129	0.02032	0.01116	0.00575	0.00789	0.02190

Streptococcus	0.01401	0.01859	0.07657	0.08239	0.00000	0.00000	0.00298	0.00342	0.02210
Ramlibacter	0.02023	0.02394	0.00323	0.00443	0.00000	0.00000	0.00000	0.00000	0.02280
Rodentibacter	0.01500	0.03353	0.07227	0.04900	0.00270	0.00603	0.00000	0.00000	0.02320
Streptomyces	0.00710	0.01589	0.02099	0.01200	0.00000	0.00000	0.00000	0.00000	0.02330
Veillonella	0.00316	0.00706	0.00568	0.00479	0.00000	0.00000	0.00000	0.00000	0.02330
Bifidobacterium	0.03257	0.02362	0.01563	0.01038	0.00371	0.00578	0.00379	0.00606	0.02360
Helicobacter	0.00000	0.00000	0.00381	0.00359	0.00804	0.00211	0.00503	0.00497	0.02510
Edaphobaculum	0.36533	0.23754	0.00813	0.01606	0.00188	0.00262	0.01445	0.03230	0.02530
Intestinimonas	0.00728	0.00972	0.01484	0.00898	0.00000	0.00000	0.00055	0.00123	0.02540
env.OPS_17_unclassified	1.55415	1.51370	0.06336	0.12440	0.02868	0.05179	0.01048	0.01912	0.02630
Mitochondria_unclassified	0.04877	0.04003	0.00079	0.00176	0.00000	0.00000	0.00945	0.01889	0.02640
Rhizobium	0.79042	0.65016	0.18267	0.29645	0.12497	0.24357	0.03056	0.05929	0.02740
Lachnospiraceae_unclassified	0.03797	0.03483	0.04667	0.02795	0.00454	0.00534	0.01584	0.00937	0.02750
Alkanindiges	0.07742	0.06256	0.03233	0.07082	0.00293	0.00654	0.00528	0.01180	0.02800
Blautia	0.00596	0.00715	0.02265	0.01331	0.00000	0.00000	0.00437	0.00634	0.03030
AAP99	2.64359	2.82013	0.20975	0.37848	0.09300	0.11008	0.06261	0.11411	0.03060
Hydrogenophaga	0.33209	0.21457	0.13218	0.15164	0.08131	0.11985	0.05547	0.11210	0.03360
Coprococcus	0.00000	0.00000	0.01053	0.00881	0.00243	0.00409	0.00082	0.00184	0.03440
Spirosomaceae_unclassified	0.26214	0.28389	0.01934	0.03308	0.00000	0.00000	0.00917	0.02050	0.03700
Shewanella	0.05688	0.11171	0.03391	0.01437	0.00719	0.00680	0.00780	0.00511	0.03810
Fimbriiglobus	1.17672	1.19344	0.12449	0.13861	1.03971	1.98544	0.04362	0.07290	0.03950
Chryseobacterium	0.19001	0.18913	0.11417	0.14953	0.02263	0.02261	0.00328	0.00603	0.04170
Saccharimonadaceae_unclassified	0.00726	0.00790	0.00217	0.00205	0.00000	0.00000	0.00000	0.00000	0.04260
Clostridium_sensu_stricto_1	0.03105	0.06942	0.13700	0.07735	0.00994	0.00870	0.01043	0.01361	0.04370
Stenotrophomonas	0.04828	0.06762	0.01387	0.01458	0.00000	0.00000	0.40849	0.90916	0.04410
Ruminococcaceae_unclassified	0.00579	0.01294	0.02686	0.01781	0.00135	0.00302	0.00051	0.00113	0.04660

Rothia	0.00868	0.01941	0.02481	0.01604	0.00000	0.00000	0.00055	0.00123	0.04760
Lactococcus	0.00000	0.00000	0.06547	0.12113	0.00108	0.00241	0.00774	0.01316	0.04830
Faecalimonas	0.00421	0.00941	0.01474	0.01017	0.00000	0.00000	0.00368	0.00517	0.04870
Kaistia	0.00000	0.00000	0.05207	0.09577	0.01460	0.03264	0.00274	0.00613	0.04910
CL500-29_marine_group	0.00676	0.00815	0.00511	0.00519	0.00000	0.00000	0.00000	0.00000	0.04910
Caproiciproducens	0.12039	0.07919	0.07592	0.05369	0.02471	0.00632	0.01318	0.01230	0.04980
Parasutterella	0.00000	0.00000	0.00296	0.00334	0.00293	0.00377	0.00000	0.00000	0.04980

Table S3 The significantly regulated pathways predicted by PICRUSt2 based on KEGG database in the pairwise comparisons of GUBF vs GUBM, GUSF vs GUSM, GUBF vs GUSF, and GUBM vs GUSM.

	GUBF vs GUBM					GUSF vs GUSM				
	Description	*P* value	GUBF	GUBM	Regulation	Description	*P* value	GUSF	GUSM	Regulation
KEGG Level 2	Energy Metabolism	0.01587	0.05612003	0.05167863	Up	Signaling Molecules and Interaction	0.00794	0.00173433	0.00127256	Up
	Immune System Diseases	0.01587	0.00049433	0.00056296	Down					
	Cancers	0.03175	0.00246251	0.00149791	Up					
	Excretory System	0.03175	0.00037128	0.00013776	Up					
	Metabolism of Other Amino Acids	0.03175	0.01736415	0.01443638	Up					

	Nervous System	0.03175	0.00082246	0.00067444	Up					
	Transcription	0.03175	0.02539882	0.02980132	Down					
	Xenobiotics Biodegradation and Metabolism	0.03175	0.02184102	0.01446042	Up					
	GUBF vs GUSF					GUBM vs GUSM				
	Description	*P* value	GUBF	GUSF		Description	*P* value	GUBM	GUSM	Regulation
	Endocrine System	0.00794	0.00356088	0.00247943	Up	Endocrine System	0.00794	0.00056296	0.00048987	Up
	Metabolism of Terpenoids and Polyketides	0.01587	0.01556185	0.01283602	Up	Immune System Diseases	0.01587	0.00287614	0.00221330	Up
	Energy Metabolism	0.03175	0.05612003	0.05190441	Up					
	GUBF vs GUBM					GUSF vs GUSM				
	Description	*P* value	GUBF	GUBM	Regulation	Description	*P* value	GUSF	GUSM	Regulation
KEGG Level 3	Retinol metabolism	0.00794	0.00052172	0.00027945	Up	Bacterial secretion system	0.00794	0.00702911	0.00562601	Up
	Biotin metabolism	0.01587	0.00142367	0.00120189	Up	D-Alanine metabolism	0.01587	0.00104054	0.00110330	Down
	Chaperones and folding catalysts	0.01587	0.01137290	0.01058467	Up	Glycerophospholipid metabolism	0.01587	0.00549683	0.00617251	Down

Ether lipid metabolism	0.01587	0.00010287	0.00002421	Up	Replication, recombination and repair proteins	0.01587	0.00651507	0.00758445	Down
Fatty acid elongation in mitochondria	0.01587	0.00000263	0.00000050	Up	Sulfur relay system	0.01587	0.00362833	0.00379967	Down
Hypertrophic cardiomyopathy (HCM)	0.01587	0.00001434	0.00000414	Up	Chronic myeloid leukemia	0.03175	0.00000238	0.00000008	Up
Metabolism of xenobiotics by cytochrome P450	0.01587	0.00163223	0.00070648	Up	Dioxin degradation	0.03175	0.00048542	0.00088915	Down
Mineral absorption	0.01587	0.00015699	0.00004372	Up	Flavone and flavonol biosynthesis	0.03175	0.00000396	0.00000052	Up
Primary immunodeficiency	0.01587	0.00048722	0.00055813		Glycerolipid metabolism	0.03175	0.00357295	0.00430907	Down
Prion diseases	0.01587	0.00014316	0.00004267	Up	Glycosaminoglycan degradation	0.03175	0.00009700	0.00002329	Up
Selenocompound metabolism	0.01587	0.00354273	0.00316006	Up	Isoflavonoid biosynthesis	0.03175	0.00000552	0.00000125	Up
Steroid biosynthesis	0.01587	0.00007045	0.00001824	Up	Lysosome	0.03175	0.00013867	0.00004018	Up
African trypanosomiasis	0.03175	0.00011058	0.00004012	Up	Notch signaling pathway	0.03175	0.00000238	0.00000008	Up
Aminobenzoate degradation	0.03175	0.00185773	0.00102128	Up	Taurine and hypotaurine metabolism	0.03175	0.00132570	0.00103568	Up

Amyotrophic lateral sclerosis (ALS)	0.03175	0.00073020	0.00024079		Wnt signaling pathway	0.03175	0.00000265	0.00000010	Up
Apoptosis	0.03175	0.00045685	0.00016034	Up	Xylene degradation	0.03175	0.00037431	0.00068375	Down
Bacterial secretion system	0.03175	0.00832668	0.00706637	Up					
Carotenoid biosynthesis	0.03175	0.00034733	0.00009047	Up					
Cell division	0.03175	0.00089443	0.00066088	Up					
Cell motility and secretion	0.03175	0.00259256	0.00181331	Up					
Chagas disease (American trypanosomiasis)	0.03175	0.00009866	0.00004113	Up					
Chlorocyclohexane and chlorobenzene degradation	0.03175	0.00058574	0.00023429	Up					
Colorectal cancer	0.03175	0.00040016	0.00012461	Up					
Drug metabolism - cytochrome P450	0.03175	0.00169868	0.00073618	Up					
Fluorobenzoate degradation	0.03175	0.00043888	0.00014806	Up					
Glutamatergic synapse	0.03175	0.00082218	0.00067402	Up					

Glycerolipid metabolism	0.03175	0.003281 22	0.003709 46	Down
Glycolysis / Gluconeogenesis	0.03175	0.009287 77	0.010107 08	Down
Influenza A	0.03175	0.000400 29	0.000124 75	Up
Inositol phosphate metabolism	0.03175	0.001353 30	0.000932 31	Up
Isoflavonoid biosynthesis	0.03175	0.000019 28	0.000005 30	Up
N-Glycan biosynthesis	0.03175	0.000206 99	0.000076 80	Up
Naphthalene degradation	0.03175	0.001554 71	0.001002 80	Up
Nitrotoluene degradation	0.03175	0.000723 81	0.000398 81	Up
Oxidative phosphorylation	0.03175	0.013558 90	0.010081 24	Up
p53 signaling pathway	0.03175	0.000400 16	0.000124 61	Up
Pathways in cancer	0.03175	0.000977 40	0.000653 29	Up
Phenylpropanoid biosynthesis	0.03175	0.000602 62	0.000281 63	Up

Polycyclic aromatic hydrocarbon degradation	0.03175	0.00025204	0.00008317	Up
Proximal tubule bicarbonate reclamation	0.03175	0.00037052	0.00013775	Up
Renin-angiotensin system	0.03175	0.00003503	0.00000627	Up
Small cell lung cancer	0.03175	0.00040016	0.00012461	Up
Steroid hormone biosynthesis	0.03175	0.00012790	0.00003643	Up
Toxoplasmosis	0.03175	0.00040025	0.00012474	Up
Tropane, piperidine and pyridine alkaloid biosynthesis	0.03175	0.00135447	0.00116182	Up
Tyrosine metabolism	0.03175	0.00328888	0.00237337	Up
Viral myocarditis	0.03175	0.00040016	0.00012461	Up

GUBF vs GUSF					GUBM vs GUSM				
Description	*P* value	GUBF	GUSF	Regulation	Description	*P* value	GUBM	GUSM	Regulation
Bacterial secretion system	0.00794	0.00832668	0.00702911	Up	Pathogenic Escherichia coli infection	0.01587	0.00001372	0.00000249	Up

Folate biosynthesis	0.00794	0.00407988	0.00335621	Up	Primary immunodeficiency	0.01587	0.00055813	0.00048937	Up
Protein digestion and absorption	0.00794	0.00010755	0.00001603	Up	Taurine and hypotaurine metabolism	0.01587	0.00125877	0.00103568	Up
Sesquiterpenoid biosynthesis	0.01116	0.00000100	0.00000003	Up	Biosynthesis of type II polyketide backbone	0.02537	0.00000019	0.00000000	Up
Adipocytokine signaling pathway	0.01587	0.00075886	0.00040160	Up	Adipocytokine signaling pathway	0.03175	0.00058939	0.00033976	Up
beta-Lactam resistance	0.01587	0.00004658	0.00001725	Up	C5-Branched dibasic acid metabolism	0.03175	0.00228228	0.00249646	Down
Carbohydrate metabolism	0.01587	0.00080324	0.00131003	Down	Dioxin degradation	0.03175	0.00046990	0.00088915	Down
Chaperones and folding catalysts	0.01587	0.01137290	0.01048971	Up	Electron transfer carriers	0.03175	0.00050613	0.00169560	Down
Fatty acid elongation in mitochondria	0.01587	0.00000263	0.00000025	Up	Flavone and flavonol biosynthesis	0.03175	0.00000428	0.00000052	Up
Hypertrophic cardiomyopathy (HCM)	0.01587	0.00001434	0.00000372	Up	Glycerolipid metabolism	0.03175	0.00370946	0.00430907	Down
Bacterial chemotaxis	0.03175	0.00697328	0.00855895	Down	Glycolysis / Gluconeogenesis	0.03175	0.01010708	0.00953906	Up
Biosynthesis of vancomycin group antibiotics	0.03175	0.00040834	0.00027716	Up	MAPK signaling pathway - yeast	0.03175	0.00055244	0.00047840	Up

Carbon fixation pathways in prokaryotes	0.03175	0.01015161	0.00858846	Up	Pyruvate metabolism	0.03175	0.01128658	0.01023171	Up
Carotenoid biosynthesis	0.03175	0.00034733	0.00009509	Up	Replication, recombination and repair proteins	0.03175	0.00673762	0.00758445	Down
Electron transfer carriers	0.03175	0.00038049	0.00084397	Down	Ribosome biogenesis in eukaryotes	0.03175	0.00053527	0.00049372	Up
Ether lipid metabolism	0.03175	0.00010287	0.00002996	Up	Stilbenoid, diarylheptanoid and gingerol biosynthesis	0.03175	0.00001296	0.00000198	Up
Flagellar assembly	0.03175	0.00891142	0.01158342	Down	Tropane, piperidine and pyridine alkaloid biosynthesis	0.03175	0.00116182	0.00129974	Down
Lipoic acid metabolism	0.03175	0.00070921	0.00058987	Up	Xylene degradation	0.03175	0.00040856	0.00068375	Down
Lysosome	0.03175	0.00032249	0.00013867	Up					
Mineral absorption	0.03175	0.00015699	0.00006457	Up					
N-Glycan biosynthesis	0.03175	0.00020699	0.00006127	Up					
Others	0.03175	0.00654849	0.00719700	Down					
Oxidative phosphorylation	0.03175	0.01355890	0.01089509	Up					

Photosynthesis - antenna proteins	0.03175	0.00012436	0.00000391	Up
PPAR signaling pathway	0.03175	0.00173792	0.00100264	Up
Prenyltransferases	0.03175	0.00224164	0.00191799	Up
Protein processing in endoplasmic reticulum	0.03175	0.00058078	0.00043126	Up
Pyruvate metabolism	0.03175	0.01118058	0.01070932	Up
Steroid biosynthesis	0.03175	0.00007045	0.00002097	Up
Systemic lupus erythematosus	0.03175	0.00000711	0.00000139	Up
Transporters	0.03175	0.05805273	0.06863945	Down

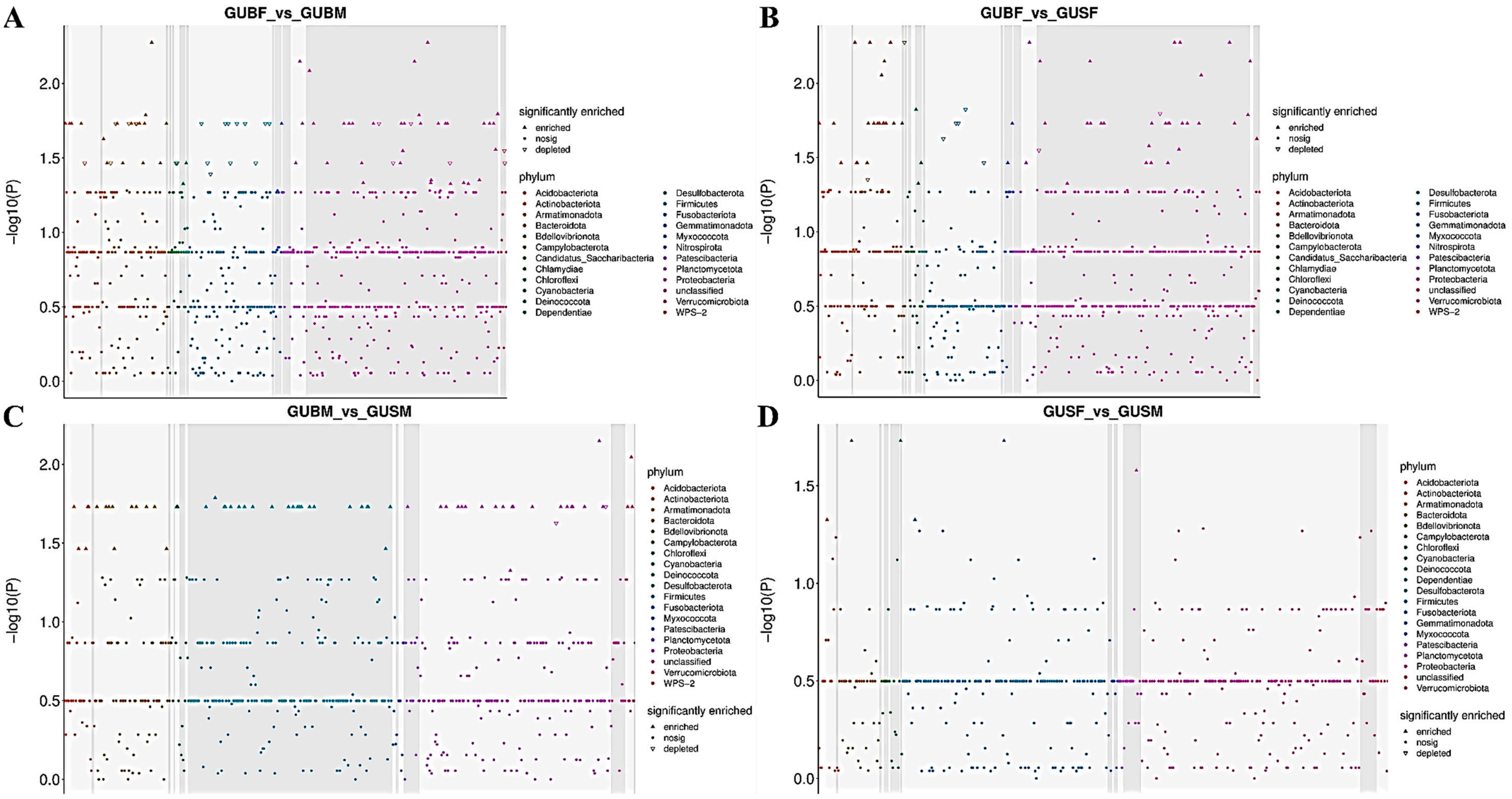

Figure S1 The Manhattan plot displaying the significantly enriched or depleted intestinal microbial phylum in abundance with a pairwise

comparison pattern. (A) The significantly enriched or depleted intestinal microbial phyla between GUBF and GUBM groups; (B) The significantly enriched or depleted intestinal microbial phyla between GUBF and GUSF groups; (C) The significantly enriched or depleted intestinal microbial phyla between GUBM and GUSM groups; (D) The significantly enriched or depleted intestinal microbial phyla between GUSF and GUSM groups.
